# Integrating Machine Learning and Dynamic Bayesian Networks to Identify the Factors Associated with Subsequent Intrapulmonary Metastasis Classification After Initial Single Primary Lung Cancer

**DOI:** 10.3390/cancers18081185

**Published:** 2026-04-08

**Authors:** Wei Liu, Aliss T. C. Chang, Joyce W. Y. Chan, Junko C. S. Chan, Rainbow W. H. Lau, Tony S. K. Mok, Calvin S. H. Ng

**Affiliations:** 1 Department of Surgery, Faculty of Medicine, The Chinese University of Hong Kong, Prince of Wales Hospital, Shatin, New Territories, Hong Kong, China; wliu@surgery.cuhk.edu.hk (W.L.); alisschang@surgery.cuhk.edu.hk (A.T.C.C.); wychan@surgery.cuhk.edu.hk (J.W.Y.C.); junkochan@surgery.cuhk.edu.hk (J.C.S.C.); rainbowlau@surgery.cuhk.edu.hk (R.W.H.L.); 2 Department of Clinical Oncology, Faculty of Medicine, The Chinese University of Hong Kong, Prince of Wales Hospital, Shatin, New Territories, Hong Kong, China; tony@clo.cuhk.edu.hk

**Keywords:** lung cancer, intrapulmonary metastasis, single primary lung cancer, random forest, dynamic Bayesian network, SEER, registry-based classification

## Abstract

**Background/Objectives:** Intrapulmonary metastasis (IPM) after an initial single primary lung cancer (SPLC) is an adverse follow-up pattern; however, when studying population-based longitudinal records, the determinants remain unclear. We aimed to identify factors associated with subsequent IPM after initial SPLC using artificial intelligence (AI)-driven analytical approaches. **Methods:** We used Surveillance, Epidemiology, and End Results (SEER) lung cancer records from 2000 to 2019. Adults with at least two records were restricted to those with SPLC at the first record. Outcome at the second record was registry-classified IPM versus persistent SPLC. A machine learning framework based on random forest models was developed using baseline variables, first record characteristics, and the interval between records. Temporal validation was performed by training on cases from 2000 to 2013 and testing on cases from 2014 to 2019. A dynamic Bayesian network (DBN) supported simulated intervention (SI) analyses to estimate model-implied risk ratios (RRs) with 95% confidence intervals (CIs). **Results:** Among 3450 patients, 361 had registry-classified IPM at the second record. The random forest model achieved an area under the curve (AUC) of 0.852 in internal validation and 0.929 in temporal validation. Surgery and record timing were the leading predictors. The DBN retained surgery as the only direct parent and achieved an AUC of 0.779. SI analyses showed higher IPM probability for pleural invasion level (PL) 3 versus PL 0, RR 1.378 (95% CI, 1.080–1.657). Lobectomy with mediastinal lymph node dissection versus wedge resection lowered the IPM probability, RR 0.378 (95% CI, 0.219–0.636). **Conclusions:** AI-based time-sequence modeling integrating machine learning and a DBN allowed for the identification of surgery, pleural invasion, and record timing as key factors associated with subsequent IPM classification after initial SPLC. This framework demonstrates the potential of combining predictive and probabilistic dependency modeling to investigate registry-based disease classification patterns, and may support hypothesis generation for future prospective studies.

## Simple Summary

Some patients with an initial lung cancer diagnosis are later classified in registry records as having additional intrapulmonary tumor involvement. This later registry-based designation is associated with worse outcomes, but the factors associated with later intrapulmonary metastasis (IPM) classification remain incompletely understood. In this study, we analyzed nationwide lung cancer records from the United States to investigate factors associated with subsequent IPM classification after an initial single lung cancer diagnosis. We applied artificial intelligence (AI) methods to analyze patient characteristics, tumor features, treatment information, and the timing between clinical records. The models identified several key factors, including surgical approach and pleural invasion, that were associated with a higher or lower likelihood of later IPM classification. These findings reveal how AI can uncover patterns in registry-based cancer data, and may support improved risk assessment and future research in lung cancer management.

## 1. Introduction

Lung cancer remains the leading cause of cancer-related mortality worldwide and continues to carry substantial long-term risk, even after apparently curative intent treatment [1]. With advances in screening, imaging, and multimodal therapy, more patients are diagnosed at an early stage and undergo surgery or other definitive management [2,3,4]; nevertheless, a clinically important proportion subsequently develop new or evolving intrathoracic disease [5,6]. In routine practice, follow-up encounters may reveal either a disease state that remains consistent with a single primary lung cancer (SPLC) or a subsequent classification consistent with intrapulmonary metastasis (IPM) [7,8,9], which usually indicates more aggressive biological behavior and worse prognosis [10,11]. Clarifying which baseline and initial visit factors are most strongly associated with this unfavorable subsequent classification is therefore important for risk stratification and for tailoring surveillance strategies.

Most prior work has examined individual prognostic factors or single modality interventions in relation to recurrence or survival [12,13,14]. However, few studies have leveraged population-level longitudinal tumor records to evaluate how baseline sociodemographic characteristics and initial clinical and treatment features jointly relate to later diagnostic classification within the same patient. In addition, conventional regression approaches often emphasize association at a single time point and may not reflect the evolving dependency structure among clinical variables, particularly when treatments and staging information are interrelated. These gaps encourage the use of methods that can support both the prediction and structured modeling of temporal dependencies while minimizing information leakage across time.

In this study, we used the Surveillance, Epidemiology, and End Results (SEER) database to identify adults with at least two lung cancer records. We restricted analyses to patients classified as SPLC at the first record and evaluated whether the subsequent record was classified as IPM or remained SPLC. To investigate the factors associated with this subsequent classification pattern, we implemented an artificial intelligence (AI)-driven analytical framework integrating machine learning and probabilistic graphical modeling. A random forest model was applied for prediction and variable importance ranking [15,16]. A dynamic Bayesian network (DBN) was then constructed to learn temporally ordered dependencies and identify proximal determinants within the learned structure [17,18,19]. Finally, simulated intervention (SI) analysis was performed to estimate model-implied contrasts in outcome probabilities under alternative values of selected first record factors [20]. This integrated AI framework aims to improve our understanding of subsequent diagnostic classification patterns after an initial SPLC diagnosis in registry data, and may help inform hypotheses regarding subsequent diagnostic classification patterns and follow-up strategies in registry-based cohorts.

## 2. Materials and Methods

### 2.1. Data Processing and Baseline Analysis

This study used the Incidence—SEER Research Data, 17 Registries, Nov 2021 sub (2000–2019) database in SEER*Stat version 8.4.4. The “Nov 2021 Sub” designation refers to the SEER data submission version, and the data were extracted in February 2025. The International Classification of Diseases for Oncology, Third Edition (ICD-O-3)/World Health Organization (WHO) 2008 was set to “Lung and Bronchus.” Inclusion criteria were (a) diagnosis between 2000 and 2019; (b) age over 18 years; (c) histologically confirmed carcinosarcoma, non-small cell adenocarcinoma, non-small cell carcinoma, non-small cell neuroendocrine carcinoma (NEC), non-small cell neuroendocrine tumors (NETs), pulmonary blastoma, small cell NEC, or unspecified malignant neoplasms except central nervous system; and (d) the patient (same patient ID) having at least two different visit records at different times. Exclusion criteria included (a) patients with malignancies before the diagnosis of primary lung cancer; and (b) incomplete information.

We organized tumor records as baseline sociodemographic variables at time zero and longitudinal clinical variables at up to four sequential records, denoted t1 through t4. Variables were harmonized across time points for age phase, stage, pathology, tumor location, treatments, and inter-record time intervals. Baseline covariates included sex, race, marital status at diagnosis, median household income, and rural–urban continuum. Because later records were sparse, analyses focused on the outcomes at the second record (i.e., t2). Patients were classified as IPM or persistent SPLC at t2, and groupwise differences were summarized.

### 2.2. Definition of IPM and SPLC

SPLC is characterized by a single primary lung tumor, while IPM occurs when tumor cells from an initial lung cancer spread within the same lung. SPLC was defined as the absence of registry-coded separate tumor nodules in the ipsilateral lung at the recorded time point. IPM was defined according to the SEER “Separate Tumor Nodules” coding framework, which classifies same-histology separate nodules in the ipsilateral lung as occurring in the same lobe, a different lobe, both same and different lobes, or an unspecified ipsilateral lobe [21]. The study outcome was defined as registry-recorded disease classification at the second record; that is, IPM versus persistent SPLC, rather than adjudicated clinical progression, recurrence, or biologically confirmed metastasis.

### 2.3. Random Forest Analysis

We trained a random forest classifier to predict the outcome at t2 (IPM versus persistent SPLC) among patients classified as SPLC at t1. For predictive modeling, we used a predefined complete-case subset after the exclusion of prespecified missing or registry-defined non-informative levels in selected predictors. These excluded levels included categories such as unknown, not assessed, not otherwise specified, or no resection of primary, depending on the variable. A participant flow diagram summarizing cohort derivation and the modeling subset is provided in Figure S1. Because these levels were heterogeneous in meaning and often reflected registry coding conventions rather than conventional randomly missing values, multiple-imputation was not performed. To improve transparency, variable-level exclusion patterns and comparisons between included and excluded patients were analyzed. Only baseline and t1 variables, together with the first inter-record time interval, were used as predictors. All year-of-diagnosis fields and all information from t2 other than the outcome label were excluded to reduce information leakage. Preprocessing, optional resampling, and model fitting were implemented within a single pipeline.

To address class imbalance and to tune the model jointly, we performed a grid search over resampling strategies, including none, random undersampling, Synthetic Minority Over-sampling Technique (SMOTE) [22,23], and SMOTE Edited Nearest Neighbors [24], as well as class weight settings. Specifically, the random forest hyperparameter grid included 400 or 600 trees, maximum depth of none, 20, or 30, minimum samples to split of 2, 5, or 10, and minimum samples per leaf of 1, 2, or 4. Class weight settings included none, balanced, and minority class upweighting ratios of 1:3, 1:5, and 1:8 relative to the majority class. Model selection was performed using macro F1.

For temporal validation, the final modeling was divided according to the year of first record into a training set from 2000 to 2013 and tested on the first records from 2014 to 2019. Internal performance was estimated using nested cross-validation within the training set, with five outer folds and three inner folds. In each outer fold, the best pipeline from the inner search was refit on the outer training data. Isotonic calibration was then fitted on the outer training data and used to generate calibrated probabilities for the held-out fold. The decision threshold was selected within the training set by maximizing F1 across a probability grid. Hyperparameter tuning, isotonic calibration, and threshold selection were performed using the training set only, and the temporal test set was used exclusively for final model evaluation. After model development was completed, the locked pipeline was fitted on the full training set and evaluated in the temporal test set. Discrimination was summarized with receiver operating characteristic (ROC) curves and area under the curve (AUC), and 95% confidence intervals (CIs) for the AUC were estimated using 2000 nonparametric bootstrap resamples of the out-of-fold predictions in the training set and temporal test predictions in the external validation set. Calibration was assessed using calibration curves together with calibration intercept and calibration slope. Overall prediction error was summarized using the Brier score. Because raw Brier values depend partly on outcome prevalence, we additionally reported the null Brier and the Brier skill score relative to a prevalence only null model. To better understand the relation between internal and temporal performance, we compared predictor distributions between the training period and the temporal validation period. For numeric predictors, between period differences were summarized using means, standard deviations, medians, interquartile ranges, and standardized mean differences. For categorical predictors, we summarized level distributions and used the maximum absolute standardized difference across levels as an overall measure of between period shift. Clinical utility was assessed with decision curve analysis (DCA) across thresholds from 0.01 to 0.99, compared with treat-all and treat-none strategies. Feature importances from the transformed design matrix were mapped back and aggregated to the original variables, and the top ten predictors are reported.

### 2.4. Dynamic Bayesian Network (DBN) Analysis

We constructed a DBN using pgmpy to model temporal dependencies among clinical variables and to predict the outcome at t2 among patients classified as SPLC at t1. The network included baseline variables, t1 variables, and time interval features, with Outcomes t2 as the only t2 variable. Structure learning used the K2 score, a Bayesian structure scoring metric that evaluates how well a candidate directed acyclic graph fits the observed discrete data given a predefined node ordering, together with greedy forward search [25,26]. The search was subject to a predefined temporal order from baseline to t1, then time intervals, then outcome. Blacklist constraints excluded clinically implausible edges. Each node was limited to a maximum of five parents, and candidate parents were added based on incremental K2 score improvement. Parameters were estimated using Bayesian estimation with a Dirichlet equivalent uniform prior.

Structural stability was assessed using 1000 bootstrap resamples with structure relearning and arc frequency summarization. Outcome neighborhood stability was evaluated by the frequency of variables in the outcome Markov blanket across bootstrap networks. Variability relative to the reference network learned from the full dataset was summarized using direction-tolerant structural Hamming distance, and Markov blanket Jaccard similarity was computed for each resample. For cross-sample validation, we relearned the structure in five folds and compared each fold to the reference using structural Hamming distance and Markov blanket Jaccard similarity. Sensitivity analyses varied parent limits and node orders consistent with temporal flow. Networks were visualized using NetworkX (version 3.3) in a time-stratified layout [27]. Predictive performance was evaluated using forward chaining folds, a temporal ordered validation strategy in which each model is trained on earlier subsets and tested on later subsets, rather than using standard random cross-validation. This design is recommended when observations have temporal dependence because random partitioning may yield optimistic estimates through temporal leakage [28,29]. We report the ROC, AUC, and Brier score.

### 2.5. Simulated Intervention (SI) Analysis

We performed SIs on the Outcomes t2 network to quantify model-implied contrasts. The network structure was fixed to the reference graph, and parameters were re-estimated using maximum likelihood to obtain conditional probability distributions. We simulated contrasts by setting intervention evidence on selected variables, such as surgery categories at t1, and compared predicted outcome probabilities under alternative interventions. Uncertainty was assessed using 1000 bootstrap resamples with parameter re-estimation in each resample. Effect sizes are summarized as risk ratios (RRs) derived from bootstrap distributions and visualized in forest plots. We report the mean, 95% CI, and two-sided *p*-value from bootstrap distributions.

### 2.6. Sensitivity Analysis

To assess the potential impact of selection bias arising from restriction to patients with a second record, we performed an additional scenario and bounds analysis in the full cohort of patients classified as SPLC at t1. This analysis included both patients with an observable t2 classification and those without a second record, for whom t2 classification was unobserved. We first calculated extreme bounds for the overall risk of subsequent IPM classification by assuming that all patients without a second record were either not classified as IPM or all classified as IPM. We then examined prespecified intermediate scenarios in which the IPM probability among patients without a second record was assigned fixed values of 1%, 3%, 5%, 10%, and 20%, or was set to 0.25, 0.50, 0.75, 1.00, 1.25, 1.50, or 2.00 times the observed IPM rate among patients with a second record. Additional stratified analyses were performed according to surgery at t1 and pleural invasion at t1.

### 2.7. Statistical Analysis

Data processing and descriptive analyses were performed in R version 4.3.2 using tidyverse (version 2.0.0) and openxlsx (version 4.2.7.1). Overall frequencies and proportions were computed for categorical variables, and group-specific distributions are summarized within outcome categories. The chi-square test was performed to assess categorical variables, and analysis of variance for continuous variables. Random forest, DBN modeling, and simulation interventions were implemented in Python version 3.12 using scikit-learn (version 1.5.1) and pgmpy (version 0.1.26). A two-sided *p*-value below 0.05 was considered statistically significant.

## 3. Results

### 3.1. Baseline Analysis

Using outcomes at the second record, patients were classified as IPM or SPLC. The analytic cohort included 3450 patients, comprising 361 with IPM and 3089 with SPLC. Clinicopathological characteristics are summarized in Tables S1–S4. Mean age at the first record was 68.37 years in the IPM group and 69.12 years in the SPLC group, with no significant difference. At the first record, significant differences between groups were primarily observed in tumor burden and treatment-related variables, including T stage, N stage, M stage, overall tumor node metastasis (TNM) stage, and surgery type (Tables S2 and S3), whereas baseline sociodemographic characteristics were largely comparable (Table S1). At the second record, differences were mainly observed in disease characteristics and treatment patterns, including pathology, tumor location, laterality, stage components, overall stage, surgery, chemotherapy, and pleural invasion (Table S4).

### 3.2. Random Forest

The cohort selection process for predictive modeling is shown in Figure S1. The baseline analytic cohort comprised 3450 patients with at least two recorded tumor events and a classifiable disease status at the second record. For predictive modeling, we restricted the cohort to 2897 patients classified as SPLC at the first record, then excluded 1135 records with prespecified missing or non-informative levels in modeling variables, leaving 1762 cases for model development. Variable-level patterns of prespecified missing or registry-defined non-informative levels are summarized in Table S5, and a comparison of patients included in and excluded from the predictive modeling subset is provided in Table S6.

**Figure 1:**
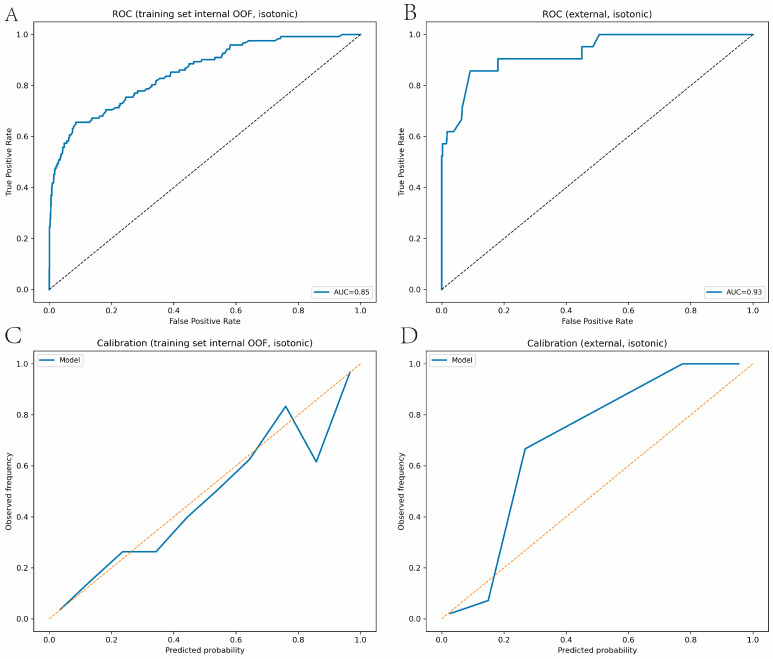
Random forest performance for predicting Outcomes_t2 among patients classified as SPLC at t1. (**A**) ROC curves in the internal training set. (**B**) ROC curves in the temporal external test set. (**C**) Calibration curve in the internal training set. (**D**) Calibration curve in the temporal external test set. The black dashed line in (**A**,**B**) represents the reference line of a non-informative classifier. The orange dashed line in (**C**,**D**) indicates perfect calibration. Isotonic refers to isotonic regression calibration, a nonparametric monotonic method used to transform model scores into calibrated predicted probabilities. Abbreviations: SPLC, single primary lung cancer; ROC, receiver operating characteristic.

The final modeling subset was divided into 1348 training cases from 2000 to 2013 and 414 test cases from 2014 to 2019. IPM prevalence was 122/1348 (9.1%) in the training set and 21/414 (5.1%) in the temporal test set. Nested cross-validation with isotonic calibration within the training set achieved an internal out-of-fold accuracy of 0.935. The threshold that maximized F1 was 0.34, yielding an IPM F1 of 0.57 with a precision of 0.72 and a recall of 0.48. In temporal validation, accuracy was 0.973, and IPM F1 was 0.65, with precision 1.00 and recall 0.48. Discrimination remained strong, with an internal AUC of 0.852 (95% CI 0.813 to 0.890) and a temporal AUC of 0.929 (95% CI 0.861 to 0.982) (Figure 1A,B). To better understand why the temporal AUC point estimate exceeded the internal estimate, we performed additional between period and performance analyses. Predictor distributions differed between the training and temporal cohorts, with the largest shift observed for the first inter record time interval, which had a standardized mean difference of 0.79. Most remaining predictors showed only small to modest between period differences, with the largest absolute standardized differences among the other variables being approximately 0.15 or less. These comparisons are summarized in Table S7.

**Figure 2:**
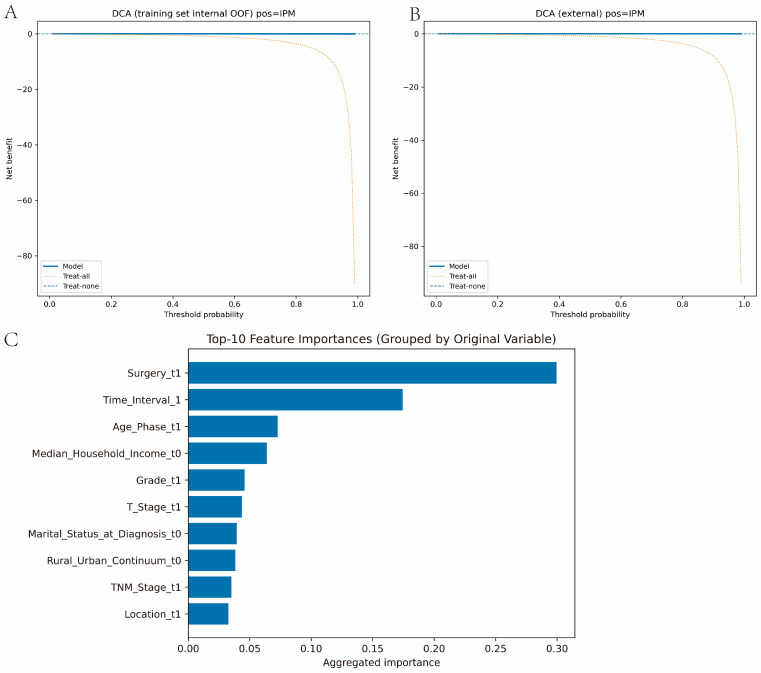
(**A**) DCA curve in the internal training set. (**B**) DCA curve in the temporal external test set. (**C**) Top predictors ranked by permutation-based variable importance from the optimized random forest model. Abbreviations: DCA, decision curve analysis.

Extended performance metrics, including average precision, Brier based measures, and calibration intercept and slope, are summarized in Table S8. The temporal cohort showed less overlap in predicted IPM probabilities between IPM and SPLC than the internal training analysis, which is consistent with easier discrimination in later years (Figure S2). Calibration was broadly reasonable but less stable at higher predicted probabilities, consistent with the smaller number of IPM events in the temporal test set (Figure 1C,D). Taken together, these analyses suggest that the higher temporal AUC point estimate likely reflects a combination of between period case mix differences, the more conservative nature of internal out of fold estimation, and sampling variability in the temporal test cohort, rather than definitive evidence that temporal validation intrinsically outperformed internal validation. The DCA showed modest positive net benefit across low to mid thresholds (Figure 2A,B). Variable importance ranked surgery at the first record the highest, followed by the first inter-record time interval and age phase. Other top predictors included median household income, grade, T stage, marital status, rural–urban continuum, overall stage, and tumor location (Figure 2C).

### 3.3. DBN

The DBN learned a reference structure with 13 edges. The outcome at the second record had a single direct parent, surgery at the first record, indicating that surgery was the most proximal determinant within the constrained search space. Other clinically coherent relations included pleural invasion directing T stage, T stage and N stage directing overall stage, and overall stage directing chemotherapy. Treatment dependencies were also observed, with chemotherapy directing systemic therapy and radiation order, and radiation order directing radiation type. Additional baseline relations included sex directing marital status and median household income directing the rural–urban continuum.

**Figure 3:**
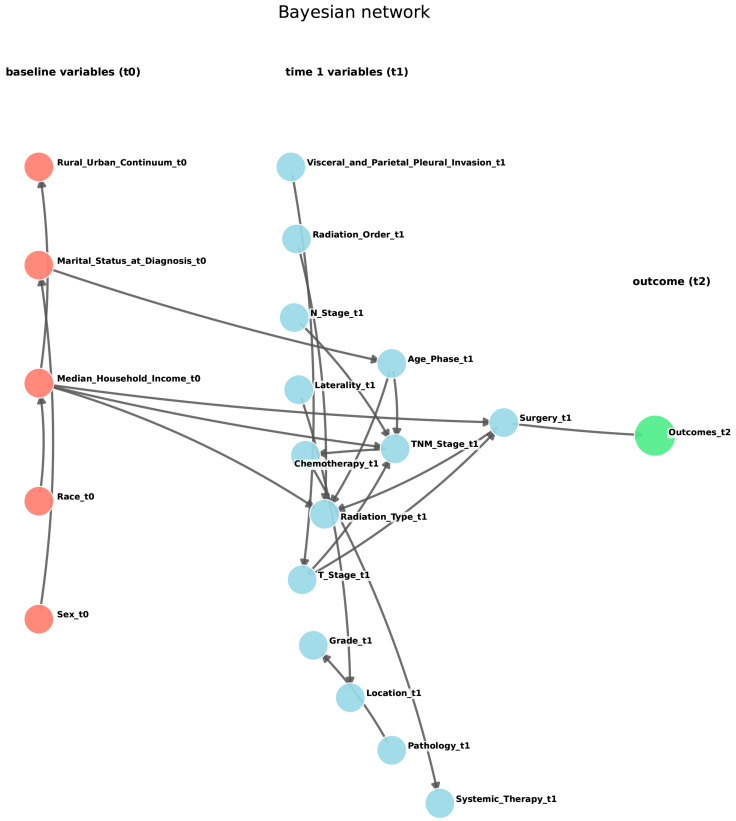
DBN-derived networks with the top 20 most stable bootstrap arcs for subsequent diagnostic classification at t2 among patients classified as SPLC at t1. Nodes are arranged by temporal layer. Pink nodes indicate baseline variables at t0, light blue nodes indicate variables at t1, and the light green node indicates the outcome at t2. Arrows represent directed dependencies learned by the DBN, pointing from parent nodes to child nodes. Abbreviations: SPLC, single primary lung cancer; DBN, Dynamic Bayesian network.

Structural stability from 1000 bootstrap resamples indicated that the edge from surgery to the outcome was perfectly stable with a frequency of 1.00. Several upstream edges also had frequency 1.00, including pleural invasion to T stage, T stage to overall stage, N stage to overall stage, overall stage to chemotherapy, and radiation order to radiation type. A candidate edge from the first-time interval to the outcome appeared often with frequency 0.778. Markov blanket analysis found that surgery was always included with frequency 1.00, and the first-time interval appeared with frequency 0.778. To preserve readability, the main network figure displays the 20 most stable bootstrap arcs (Figure 3). An extended network including all arcs with a bootstrap frequency of at least 0.50 is provided in Figure S3. Edge-level variability measured by structural Hamming distance had a median of 13 with an interquartile range of 10 to 16. In five-fold cross-validation, the structural Hamming distance ranged from 2 to 16 with a median of 7, and the outcome Markov blanket Jaccard similarity was 1.00 in all folds. Out-of-fold prediction yielded an area under the curve of 0.779 (Figure S4).

### 3.4. SIs of Clinicopathological Characteristics

**Figure 4:**
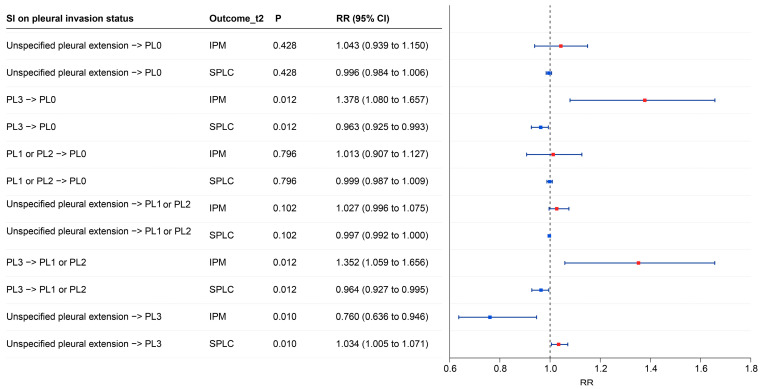
Model-implied RRs for Outcomes_t2 under SIs on visceral and parietal pleural invasion at t1. RRs are presented as the predicted outcome probability under the first listed category relative to that under the second listed category. In the forest plot, square markers indicate point estimates of the RRs, horizontal lines indicate 95% CIs derived from bootstrap resampling, and the vertical dashed line marks the null value of RR = 1. Red squares denote IPM and blue squares denote SPLC. Abbreviations: RR, risk ratio; SI, simulated intervention; CI, confidence interval; PL, pleural invasion level.

SIs suggested that pleural invasion at the first record was the only evaluated clinicopathological factor with consistent evidence of association with the outcome under the current specification (Figure 4), whereas sex, age, grade, and stage showed no supported shifts (Figure S5). Contrasts involving pleural invasion level (PL) 3 showed the largest estimated differences in outcome probability. Compared with PL0, PL3 was associated with a higher model-implied probability of IPM and a lower probability of SPLC, with RR 1.378 (95% CI 1.080 to 1.657) for IPM and 0.963 (95% CI 0.925 to 0.993) for SPLC. Similar results were observed for PL3 versus PL1 or PL2, with RR 1.352 (95% CI 1.059 to 1.656) and 0.964 (95% CI 0.927 to 0.995), respectively. Compared with PL3, unspecified pleural extension was associated with a lower model-implied probability of IPM and a higher probability of SPLC, with RR 0.760 (95% CI 0.636 to 0.946) and 1.034 (95% CI 1.005 to 1.071), respectively.

### 3.5. SIs of Surgery

**Figure 5:**
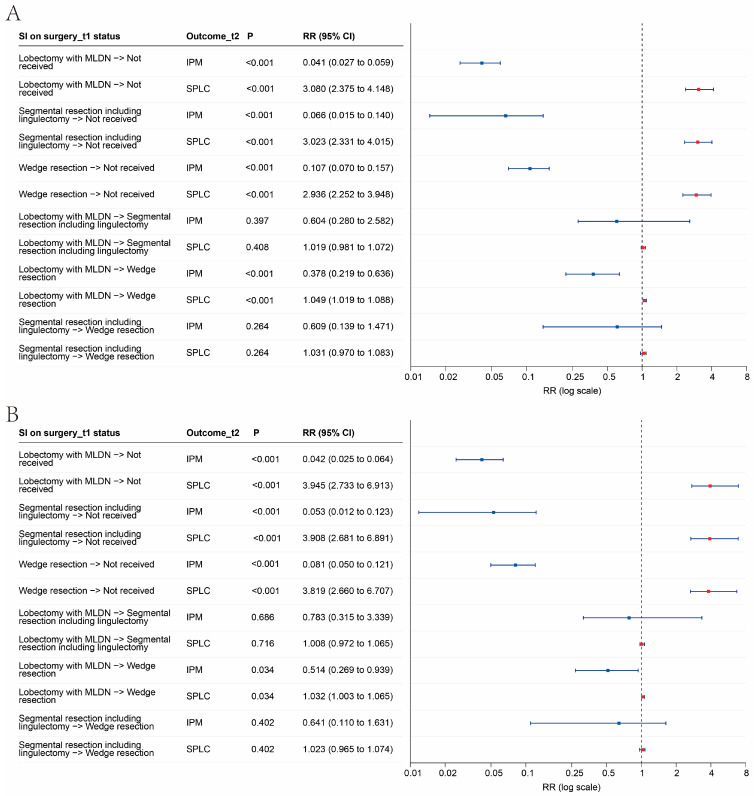
Model-implied RRs for Outcomes_t2 under SIs on surgery at t1. (**A**) Overall cohort. (**B**) Stage I subgroup. RRs are presented as the predicted outcome probability under the first listed category relative to that under the second listed category. In the forest plots, square markers indicate point estimates of the RRs, horizontal lines indicate 95% CIs derived from bootstrap resampling, and the vertical dashed line marks the null value of RR = 1. Red squares denote IPM and blue squares denote SPLC. Both panels use the same logarithmic *x*-axis scale to facilitate direct visual comparison across contrasts with widely differing effect sizes and confidence interval widths, and exact RR estimates with 95% CIs are shown in the accompanying table. Abbreviations: RR, risk ratio; SI, simulated intervention; CI, confidence interval.

Simulation interventions related to surgery demonstrated consistent patterns in the overall cohort and in stage I disease, as shown in Figure 5A,B. Across all TNM stages, shifting from lobectomy with mediastinal lymph node dissection (MLND) to not-received was associated with a markedly lower estimated risk of IPM and a higher estimated risk of SPLC, with RR 0.041 (95% CI 0.027–0.059, *p* < 0.001) for IPM and RR 3.080 (95% CI 2.375–4.148, *p* < 0.001) for SPLC. Similar directions were observed for other surgical categories compared with not-received. Specifically, segmental resection including lingulectomy to not-received yielded RR 0.066 (0.015–0.140, *p* < 0.001) for IPM and RR 3.023 (2.331–4.015, *p* < 0.001) for SPLC, while wedge resection to not-received yielded RR 0.107 (0.070–0.157, *p* < 0.001) for IPM and RR 2.936 (2.252–3.948, *p* < 0.001) for SPLC. Comparisons between surgical procedures were more heterogeneous. Notably, lobectomy with MLND compared to wedge resection was associated with a lower estimated risk of IPM and a modestly higher estimated risk of SPLC, with RR 0.378 (0.219–0.636, *p* < 0.001) for IPM and RR 1.049 (1.019–1.088, *p* < 0.001) for SPLC, whereas lobectomy to segmental resection and segmental resection to wedge resection were not statistically supported.

Stage I subgroup analyses showed concordant findings with clearer clinical interpretation (Figure 5B). Compared with not-received, lobectomy with MLND was associated with RR 0.042 (95% CI 0.025–0.064, *p* < 0.001) for IPM and RR 3.945 (2.733–6.913, *p* < 0.001) for SPLC. Segmental resection including lingulectomy and wedge resection compared with not-received also demonstrated reduced IPM and increased SPLC, with RR 0.053 (0.012–0.123, *p* < 0.001) and RR 3.908 (2.681–6.891, *p* < 0.001) for segmental resection, and RR 0.081 (0.050–0.121, *p* < 0.001) and RR 3.819 (2.660–6.707, *p* < 0.001) for wedge resection. Among procedure-to-procedure contrasts in stage I, lobectomy to wedge resection remained statistically supported, with RR 0.514 (0.269–0.939, *p* = 0.034) for IPM and RR 1.032 (1.003–1.065, *p* = 0.034) for SPLC, whereas comparisons involving segmental resection versus wedge resection were not statistically supported. In the learned network, adjuvant therapies at the first record, including chemotherapy, systemic therapy, radiation type, and radiation order, did not form any direct or indirect path to the outcome at the second record, suggesting no modeled influence of adjuvant therapy on the subsequent outcome classification under the current specification.

### 3.6. Selection Bias Sensitivity Analysis

Among 37,700 patients classified as SPLC at t1, 2897 had an observable t2 classification and 34,803 had no second record. In the observed subset, 206 patients were classified as IPM at t2, corresponding to an observed rate of 7.11%. Because 92.3% of the cohort lacked a second record, the estimated overall IPM risk was highly sensitive to assumptions about outcomes in the missing group. Under the extreme lower bound, the overall IPM rate was 0.55%, whereas under the extreme upper bound it was 92.86% (Table S9). In intermediate scenario analyses, the overall IPM rate ranged from 1.47% to 19.01% when the assumed IPM probability among patients without a second record was fixed between 1% and 20%. When the assumed probability was set to 0.50, 1.00, and 1.50 times the observed rate, the corresponding overall IPM rates were 3.83%, 7.11%, and 10.39%, respectively (Table S10).

In stratified analyses, surgery at t1 showed the clearest and most consistent pattern. In the observed subset, the IPM rate was 16.39% among patients who did not receive surgery, 8.03% after wedge resection, and 2.63% after lobectomy with mediastinal lymph node dissection. This ordering was preserved across within stratum multiplier scenarios, although group differences became less pronounced when a common fixed IPM probability was assigned to all patients without a second record (Tables S11 and S12). Pleural invasion categories also showed heterogeneous observed rates in the scenario analysis, but the pattern was less stable than that observed for surgery under alternative missing outcome assumptions. Consistent with the simulated intervention analysis, PL3 appeared to represent a higher risk category, whereas PL0 and PL1 or PL2 were more similar to each other. The category of tumor extension to pleura not otherwise specified showed intermediate behavior across analyses (Tables S13 and S14). Overall, these findings indicate that the absolute population risk of subsequent IPM classification depends strongly on assumptions regarding patients without a second record, whereas the main directional signal for surgery remains broadly consistent across a range of plausible scenarios.

## 4. Discussion

Using SEER longitudinal tumor records, we examined subsequent diagnostic classification at the second record among patients classified as SPLC at the first record. We integrated a random forest model for prediction and feature ranking, a DBN to characterize temporally ordered dependencies, and SI analyses to quantify model-implied contrasts in outcome probabilities under alternative values of selected first record factors. Across methods, surgery at the first record emerged as the most proximal variable associated with the second record classification within the constrained search space, while the random forest highlighted a broader set of predictors that included surgery, time interval, age phase, and several socioeconomic and staging-related variables. These results support the view that the subsequent classification at the second record is shaped by a combination of tumor burden, treatment selection, and follow-up intensity, rather than by a single clinicopathological factor [30,31,32].

SI analyses supported two main patterns. First, among the evaluated clinicopathological variables, pleural invasion related contrasts involving PL3 showed the clearest model-implied shifts in outcome probability. This aligns with the clinical expectation that pleural invasion reflects more locally invasive tumor behavior and is associated with higher risks of postoperative recurrence, including local recurrence patterns, which is compatible with a greater propensity for intrathoracic or ipsilateral disease manifestation [33,34,35]. Second, surgery-related SI scenarios produced large shifts in the estimated distribution of the second record classification. In both the overall cohort and the stage I subgroup, SI comparisons between surgical categories and no surgery were associated with a lower estimated probability of IPM and a higher estimated probability of SPLC. These results should be interpreted carefully. Our analytic cohort was restricted to patients with at least two recorded tumor events in the SEER database, as the study objective required evaluation of disease classification at a subsequent record. In SEER, patients with only a single recorded tumor event provide information on survival status and survival time but lack information on disease state at follow-up, such as recurrence, progression, or intrapulmonary metastasis. Therefore, these patients could not contribute to the analysis of disease state transitions and were not eligible for inclusion. Accordingly, the estimates describe shifts within a cohort with observed subsequent disease classification rather than absolute risks of cure or remaining disease-free. This selection mechanism may enrich the analytic cohort for patients with greater tumor burden, more intensive surveillance, or treatment pathways associated with re-detection of disease, and therefore may influence the apparent importance of some determinants, particularly treatment-related variables. It may also introduce selection-induced collider bias because inclusion in the analytic cohort depended on having a subsequent recorded disease classification. Moreover, a higher probability of SPLC within this selected cohort does not imply better overall outcomes in the full treated population. Instead, it indicates that, conditional on having a subsequent recorded disease classification, the model allocates probability mass between a more favorable recorded state and a less favorable recorded state. A previous report also showed that the probability of second primary events increased after resection regardless of procedure type [36].

Our findings suggest that, among patients classified as SPLC at the first record and with a subsequent recorded disease classification, lobectomy with MLND was associated with a lower probability of IPM classification than wedge resection. This may reflect differences in oncologic adequacy and nodal assessment [37,38]. However, given the selected nature of the analytic cohort, potential differential follow-up, confounding by indication, and unmeasured confounding in registry data, this result should not be interpreted as direct evidence that lobectomy reduces the absolute risk of subsequent IPM classification more than wedge resection in the full initial SPLC population. Rather, it should be regarded as a hypothesis-generating signal that warrants validation in cohorts with imaging-based follow-up, recurrence adjudication, and more complete longitudinal clinical information.

Although our SEER-based dataset is large and population-representative, several limitations warrant emphasis. First, SEER tumor records do not capture disease-free status, imaging response, or detailed recurrence timing between records, so transitions from SPLC to IPM should be interpreted as changes in recorded diagnostic classification rather than confirmed clinical progression or recurrence. Second, SEER lacks key clinical detail, including comorbidities, functional status, smoking history, targeted therapy and immunotherapy, and granular treatment information such as dose, regimen, and timing. In addition, the study period from 2000 to 2019 spans major changes in lung cancer staging and management, including the implementation of the seventh edition TNM staging system in 2010 and delayed implementation of the eighth edition in the United States until January 1, 2018 [39,40]. Molecular testing recommendations expanded during the targeted therapy era, as reflected in the 2013 and 2018 guideline updates [41,42]. First-line immune checkpoint therapy also entered routine management beginning in 2016 [43]. Therefore, associations learned from pooled data across this period may partly reflect cross-era differences in staging definitions, diagnostic work-up, and treatment selection, rather than a fully time-invariant clinical process. Third, later records were sparse, and missingness increased with record number, which constrained modeling beyond the second record. Fourth, the temporal split provides a useful within-SEER validation design, but it does not replace validation across treatment eras or external validation in an independent non-SEER dataset. Accordingly, the present findings should not be assumed to generalize directly to contemporary immunotherapy era management or to healthcare systems outside the United States. In addition, the predictive modeling subset was derived using a predefined complete-case approach after exclusion of registry-defined missing or non-informative levels. Because many of these excluded levels reflected heterogeneous registry coding categories rather than conventional randomly missing values, imputation was not performed; however, this approach may introduce additional selection bias, and the characteristics of included and excluded patients are now reported for transparency. Finally, the DBN structure learning approach is sensitive to variable availability, discretization, and imposed constraints, and unmeasured confounding may induce spurious dependencies or obscure true pathways. This concern is particularly relevant for surgery-related contrasts because treatment assignment in observational registry data is nonrandom and may be influenced by confounding by indication, including factors such as comorbidity, functional status, operability, and physician decision-making, which are not fully captured in SEER.

## 5. Conclusions

Among patients classified as SPLC at the first record and with a subsequent recorded disease classification at the second record, an integrated analytical framework combining random forest prediction, DBN modeling, and SI analysis identified surgery at the first record as the most proximal variable associated with subsequent diagnostic classification within the learned structure. Pleural invasion and record timing were also associated with the subsequent IPM classification. These findings provide signals for risk stratification and generate prioritized hypotheses for future prospective studies in cohorts with richer clinical, imaging, and treatment information. More broadly, this study illustrates an AI-enabled framework for modeling temporally ordered dependencies in population-based cancer registries.

## Data Availability

The data for this article are publicly available at: https://seer.cancer.gov/ (accessed on 23 February 2025).

## Supplementary Materials

The following supporting information can be downloaded at https://www.mdpi.com/article/10.3390/cancers18081185/s1. Figure S1: Flowchart of patient selection and analytic cohorts; Table S1: Baseline and sociodemographic characteristics of patients classified at the second record (t2) as IPM or SPLC; Table S2: Tumor characteristics at the first record (t1) among patients classified at the second record (t2) as IPM or SPLC; Table S3: Treatment characteristics and pleural invasion status at the first record (t1) among patients classified at the second record (t2) as IPM or SPLC; Table S4: Tumor characteristics and treatment at the second record (t2) among patients classified at the second record (t2) as IPM or SPLC; Table S5: Prespecified missing or registry-defined non-informative levels in candidate modeling variables among patients classified as SPLC at the first record (*n* = 2897); Table S6: Comparison of patients included in and excluded from the predictive modeling subset among those classified as SPLC at the first record; Table S7: Comparison of predictor distributions between the training cohort and the temporal test cohort for the random forest analysis; Table S8: Extended performance metrics of the random forest model in the internal training analysis and temporal test cohort; Figure S2: Distribution of predicted probabilities for IPM generated by the random forest model in the internal training analysis and the temporal validation cohort. (A) In the internal training analysis, probabilities were based on nested cross validation out of fold predictions. (B) In the temporal validation cohort, probabilities were generated by applying the final calibrated model trained on the full development cohort to later years; Figure S3: Extended Bayesian network including all arcs with bootstrap frequency ≥ 0.50. Nodes are arranged by temporal layer. Pink nodes indicate baseline variables at t0, light blue nodes indicate variables at t1, and the light green node indicates the outcome at t2. Solid arrows represent the directed dependencies shown in the learned network; Figure S4: ROC curves for predicting Outcomes_t2 using forward chaining evaluation; Figure S5: Model-implied RRs for Outcomes_t2 under SIs on selected clinicopathological variables at t1. Forest plots show RRs and 95% CIs derived from bootstrap resampling; Table S9: Overall bounds summary for subsequent IPM classification among patients with initial SPLC; Table S10: Overall scenario analysis for subsequent IPM classification among patients with initial SPLC; Table S11: Stratified bounds and fixed probability scenario analysis by surgery at t1 among patients with initial SPLC; Table S12: Stratified multiplier scenario analysis by surgery at t1 among patients with initial SPLC; Table S13: Stratified bounds and fixed probability scenario analysis by pleural invasion at t1 among patients with initial SPLC; Table S14. Stratified multiplier scenario analysis by pleural invasion at t1 among patients with initial SPLC.

## Abbreviations

The following abbreviations are used in this manuscript:

AI	Artificial Intelligence
AUC	Area Under the Curve
CI	Confidence Interval
DBN	Dynamic Bayesian Network
DCA	Decision Curve Analysis
F1	F1 Score
ICD-O-3	International Classification of Diseases for Oncology, Third Edition
IPM	Intrapulmonary Metastasis
MLND	Mediastinal Lymph Node Dissection
NEC	Neuroendocrine Carcinoma
NET	Neuroendocrine Tumor
NOS	Not Otherwise Specified
NSCA	Non-Small Cell Adenocarcinoma
PL	Pleural Invasion Level
ROC	Receiver Operating Characteristic
RR	Risk Ratio
SEER	Surveillance, Epidemiology, and End Results
SI	Simulated Intervention
SMOTE	Synthetic Minority Over-sampling Technique
SPLC	Single Primary Lung Cancer
TNM	Tumor Node Metastasis
WHO	World Health Organization
