# Supplementary material for: Integrating Machine Learning and Dynamic Bayesian Networks to Identify the Factors Associated with Subsequent Intrapulmonary Metastasis Classification After Initial Single Primary Lung Cancer
Source: Cancers (Basel). 2026 Apr 8;18(8):1185. doi: 10.3390/cancers18081185 (PMC13114358; doi:10.3390/cancers18081185)
Supplement: Supplementary file 1 [file cancers-18-01185-s001.zip › cancers-4220874-supplementary.pdf]

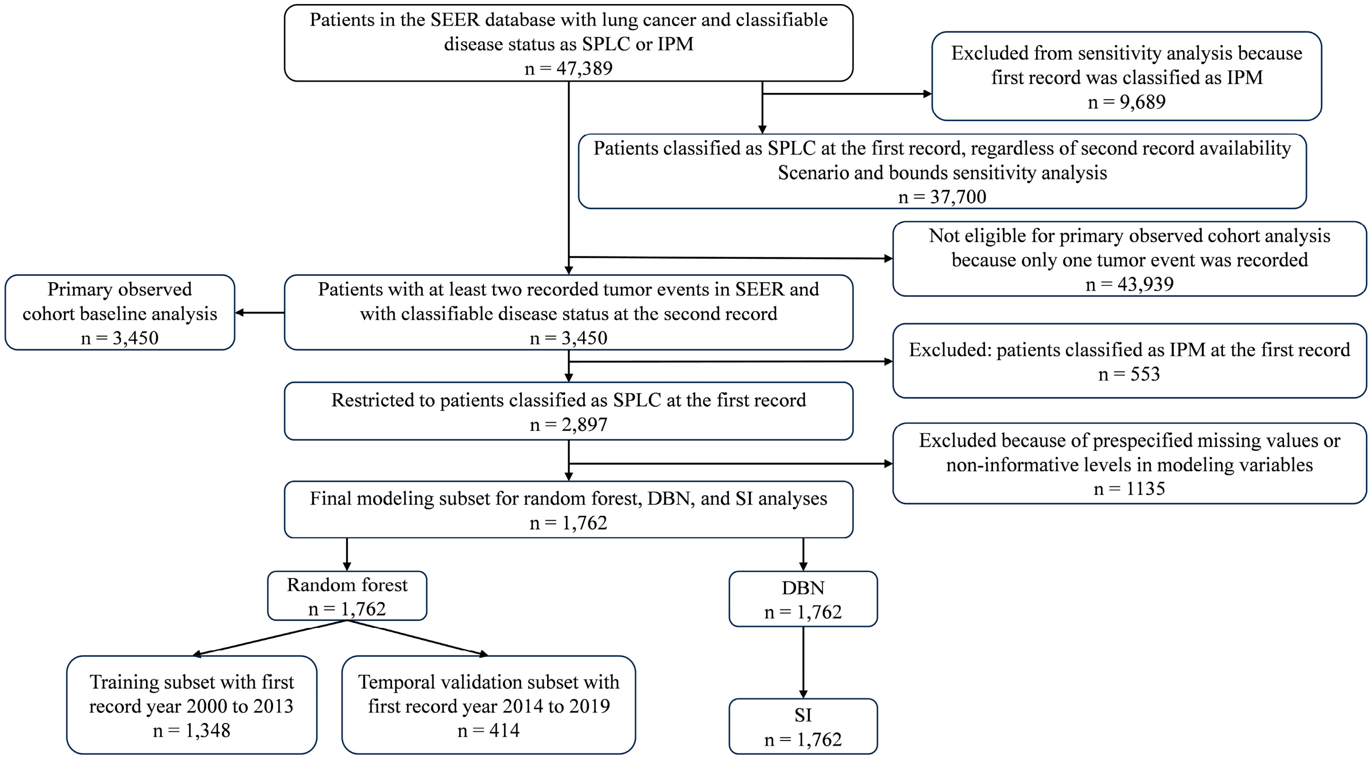

Figure S1. Flowchart of patient selection and analytic cohorts. Abbreviations: IPM, intrapulmonary metastasis; SPLC, single primary lung cancer; DBN, dynamic Bayesian network; SI , simulated intervention.

Table S1. Baseline and sociodemographic characteristics of patients classified at the second record (t2) as IPM or SPLC.

Characteristics	Cohort N=3450	IPM N=361	SPLC N=3089	P-value
Age at first record (t1), year, mean ± SD	69.04 ± 8.61	68.37 ± 9.16	69.12 ± 8.54	0.138
Sex at baseline (t0), No. (%)				0.815
Female	1877 (54.41%)	199 (55.12%)	1678(54.32%)	
Male	1573 (45.59%)	162 (44.88%)	1411 (45.68%)	
Race at baseline (t0), No. (%)				0.561
American Indian/Alaska Native	13 (0.38%)	1 (0.28%)	12 (0.39%)	
Asian or Pacific Islander	185 (5.36%)	25 (6.93%)	160 (5.18%)	
Black	265 (7.68%)	28 (7.76%)	237 (7.67%)	
White	2987 (86.58%)	307 (85.04%)	2680 (86.76%)	
Marital status at diagnosis (t0), No. (%)				0.515
Divorced	461 (13.36%)	40 (11.08%)	421 (13.63%)	
Married (including common law)	1902 (55.13%)	200 (55.4%)	1702 (55.1%)	
Separated	41 (1.19%)	4 (1.11%)	37 (1.2%)	
Single (never married)	357 (10.35%)	39 (10.8%)	318 (10.29%)	
Unmarried or Domestic Partner	7 (0.2%)	0	7 (0.23%)	
Widowed	539 (15.62%)	57 (15.79%)	482 (15.6%)	
Missing	143 (4.14%)	21 (5.82%)	122 (3.95%)	
Median household income (t0), No. (%)				0.671
<35,000	75 (2.17%)	10 (2.77%)	65 (2.1%)	
35,000-39,999	120 (3.48%)	13 (3.6%)	107 (3.46%)	
40,000-44,999	173 (5.01%)	17 (4.71%)	156 (5.05%)	
45,000-49,999	207 (6%)	25 (6.93%)	182 (5.89%)	
50,000-54,999	287 (8.32%)	34 (9.42%)	253 (8.19%)	
55,000-59,999	273 (7.91%)	24 (6.65%)	249 (8.06%)	
60,000-64,999	631 (18.29%)	76 (21.05%)	555 (17.97%)	
65,000-69,999	436 (12.64%)	47 (13.02%)	389 (12.59%)	
70,000-74,999	201 (5.83%)	20 (5.54%)	181 (5.86%)	
>75,000	1047 (30.35%)	95 (26.32%)	952 (30.82%)	
Rural–urban continuum (t0), No. (%)				0.438
Counties in metropolitan areas of 1 million people or more	1964 (56.93%)	209 (57.89%)	1755 (56.81%)	
Counties in metropolitan areas of 250,000 to 1 million people	750 (21.74%)	72 (19.94%)	678 (21.95%)	

Counties in metropolitan areas of less than 250 thousand people	248 (7.19%)	21 (5.82%)	227 (7.35%)
Nonmetropolitan counties adjacent to a metropolitan area	265 (7.68%)	36 (9.97%)	229 (7.41%)
Nonmetropolitan counties not adjacent to a metropolitan area	220 (6.38%)	23 (6.37%)	197 (6.38%)
Missing	3 (0.09%)	0	3 (0.1%)

Footnote: Values are presented as No. (%) unless otherwise indicated. Continuous variables are presented as mean ± standard deviation. P values were calculated using the chi square test for categorical variables and analysis of variance for continuous variables. Percentages may not sum to 100 because of rounding. Abbreviations: IPM, intrapulmonary metastasis; SPLC, single primary lung cancer; SD, standard deviation.

Table S2. Tumor characteristics at the first record (t1) among patients classified at the second record (t2) as IPM or SPLC.

Characteristics	Cohort N=3450	IPM N=361	SPLC N=3089	P-value
Pathology at first record (t1), No. (%)				0.276
Carcinosarcoma	1 (0.03%)	0	1 (0.03%)	
Non-small cell NEC	53 (1.54%)	3 (0.83%)	50 (1.62%)	
Non-small cell NET	59 (1.71%)	3 (0.83%)	56 (1.81%)	
Non-small cell adenocarcinoma	2215 (64.2%)	252 (69.81%)	1963 (63.55%)	
Non-small cell carcinoma	1056 (30.61%)	95 (26.32%)	961 (31.11%)	
Pulmonary blastoma	1 (0.03%)	0	1 (0.03%)	
Small cell NEC	61 (1.77%)	8 (2.22%)	53 (1.72%)	
Unspecified malignant neoplasms except CNS	4 (0.12%)	0	4 (0.13%)	
Tumor location at first record (t1), No. (%)				0.156
Lower lobe	1105 (32.03%)	112 (31.02%)	993 (32.15%)	
Middle lobe	196 (5.68%)	22 (6.09%)	174 (5.63%)	
Upper lobe	2046 (59.3%)	212 (58.73%)	1834 (59.37%)	
Main bronchus	20 (0.58%)	1 (0.8%)	19 (0.62%)	
Overlapping lesion of lung	22 (0.64%)	6 (1.66%)	16 (0.52%)	
Missing	61 (1.77%)	8 (2.22%)	53 (1.72%)	
Grade at first record (t1), No. (%)				0.048
Well-differentiated	612 (17.74%)	78 (21.61%)	534 (17.29%)	
Moderately differentiated	1508 (43.71%)	162 (44.88%)	1346 (43.57%)	
Poorly differentiated	1248 (36.17%)	110 (30.47%)	1138 (36.84%)	
Undifferentiated	82 (2.38%)	11 (3.05%)	71 (2.3%)	
Laterality at first record (t1), No. (%)				0.597

Bilateral	1 (0.03%)	0	0	
Left	1505 (43.62%)	149 (41.27%)	1357 (43.9%)	
Right	1944 (56.35%)	212 (58.73%)	1732 (56.07%)	
TNM stage at first record (t1), No. (%)				<0.001
IA	1156 (33.51%)	86 (23.82%)	1070 (34.64%)	
IB	589 (17.07%)	53 (14.68%)	536 (17.35%)	
IINOS	2 (0.06%)	0	2 (0.06%)	
IIA	240 (6.96%)	16 (4.43%)	224 (7.25%)	
IIB	325 (9.42%)	77 (21.33%)	248 (8.03%)	
IIINOS	10 (0.29%)	0	10 (0.32%)	
IIIA	360 (10.43%)	51 (14.13%)	309 (10%)	
IIIB	54 (1.57%)	7 (1.94%)	47 (1.52%)	
IV	182 (5.28%)	41 (11.36%)	141 (4.56%)	
NOS	34 (0.99%)	5 (1.39%)	29 (0.94%)	
Missing	498 (14.43%)	25 (6.93%)	473 (15.31%)	
T stage at first record (t1), No. (%)				<0.001
T1NOS	1 (0.03%)	0	1 (0.03%)	
T1a	848 (24.58%)	61 (16.9%)	787 (25.48%)	
T1b	452 (13.1%)	31 (8.59%)	421 (13.63%)	
T2NOS	15 (0.43%)	1 (0.28%)	14 (0.45%)	
T2a	742 (21.51%)	65 (18.01%)	677 (21.92%)	
T2b	160 (4.64%)	13 (3.6%)	147 (4.76%)	
T3	482 (13.97%)	106 (29.36%)	376 (12.17%)	
T4	215 (6.23%)	54 (14.96%)	161 (5.21%)	
TNOS	37 (1.07%)	5 (1.39%)	32 (1.03%)	
Missing	499 (14.46%)	25 (6.93%)	474 (15.34%)	
N stage at first record (t1), No. (%)				<0.001
N0	2397 (69.48%)	276 (76.45%)	2121 (68.66%)	
N1	229 (6.64%)	27 (7.48%)	202 (6.54%)	
N2	262 (7.59%)	21 (5.82%)	241 (7.8%)	
N3	44 (1.28%)	10 (2.77%)	34 (1.1%)	
NX	20 (0.58%)	2 (0.55%)	18 (0.58%)	
Missing	499 (14.46%)	25 (6.93%)	474 (15.34%)	
M stage at first record (t1), No. (%)				<0.001

M0	2769 (80.26%)	295 (81.72%)	2474 (80.09%)
M1a	106 (3.07%)	24 (6.65%)	82 (2.65%)
M1b	76 (2.2%)	17 (4.71%)	59 (1.91%)
Missing	499 (14.46%)	25 (6.93%)	474 (15.34%)

Footnote: Values are presented as No. (%) unless otherwise indicated. P values were calculated using the chi square test. Percentages may not sum to 100 because of rounding. Abbreviations: IPM, intrapulmonary metastasis; SPLC, single primary lung cancer; NEC, neuroendocrine carcinoma; NET, neuroendocrine tumor; CNS, central nervous system; NOS, not otherwise specified; TNM, tumor node metastasis.

Table S3. Treatment characteristics and pleural invasion status at the first record (t1) among patients classified at the second record (t2) as IPM or SPLC.

**Characteristics**	**Cohort N=3450**	**IPM N=361**	**SPLC N=3089**	**P-value**
Surgery at first record (t1), No. (%)				<0.001
Bronchial sleeve resection ONLY	1 (0.03%)	0	1 (0.03%)	
Electrocautery	1 (0.03%)	0	1 (0.03%)	
Excision	4 (0.12%)	2 (0.55%)	2 (0.06%)	
Excision or resection of less than one lobe	8 (0.23%)	0	8 (0.26%)	
Extended pneumonectomy plus pleura or diaphragm	2 (0.06%)	0	2 (0.06%)	
Laser ablation or cryosurgery	9 (0.26%)	1 (0.28%)	8 (0.26%)	
Laser excision	1 (0.03%)	0	1 (0.03%)	
Lobe or bilobectomy extended	48 (1.39%)	4 (1.11%)	44 (1.42%)	
Lobe or bilobectomy extended WITH diaphragm	1 (0.03%)	0	1 (0.03%)	
Lobe or bilobectomy extended WITH pericardium	1 (0.03%)	0	1 (0.03%)	
Lobe or bilobectomy extended chest wall	14 (0.41%)	2 (0.55%)	12 (0.39%)	
Lobectomy WITH MLND	1476 (42.78%)	91 (25.21%)	1385 (44.84%)	
Pneumonectomy	17 (0.49%)	2 (0.55%)	15 (0.49%)	
Radiofrequency ablation	1 (0.03%)	0	1 (0.03%)	
Resection of lobe or bilobectomy but less than the whole lung	279 (8.09%)	23 (6.37%)	256 (8.29%)	
Resection of lung but NOS	2 (0.06%)	0	2 (0.06%)	
Segmental resection including lingulectomy	160 (4.64%)	12 (3.32%)	148 (4.79%)	
Surgery but NOS	2 (0.06%)	0	2 (0.06%)	
WITH mediastinal lymph node dissection (radical pneumonectomy)	34 (0.99%)	5 (1.39%)	29 (0.94%)	
Wedge resection	581 (16.84%)	70 (19.39%)	511 (16.54%)	

Not-received	804 (23.3%)	148 (41.00%)	656 (21.24%)	
Missing	4 (0.12%)	1 (0.28%)	3 (0.1%)	
Radiation order at first record (t1), No. (%)				0.928
Intraoperative radiation	3 (0.09%)	0	3 (0.1%)	
Radiation after surgery	244 (7.07%)	25 (6.93%)	219 (7.09%)	
Radiation before and after surgery	4 (0.12%)	1 (0.28%)	3 (0.1%)	
Radiation prior to surgery	25 (0.72%)	3 (0.83%)	22 (0.71%)	
Sequence unknown but both were given	2 (0.06%)	0	2 (0.06%)	
Surgery both before and after radiation	3 (0.09%)	0	3 (0.1%)	
No radiation and/or cancer-directed surgery	3169 (91.86%)	332 (91.97%)	2837 (91.84%)	
Radiation type at first record (t1), No. (%)				0.480
Beam radiation	673 (19.51%)	57 (15.79%)	616 (19.94%)	
Radiation but NOS method or source not specified	10 (0.29%)	1 (0.28%)	9 (0.29%)	
Radioactive implants including brachytherapy	8 (0.23%)	0	8 (0.26%)	
Radioisotopes	1 (0.03%)	0	1 (0.03%)	
Recommended, unknown if administered	36 (1.04%)	5 (1.39%)	31 (1%)	
None/Unknown	2705 (78.41%)	297 (82.27%)	2408 (77.95%)	
Refused	17 (0.49%)	1 (0.28%)	16 (0.52%)	
Chemotherapy at first record (t1), No. (%)				0.381
Yes	961 (27.86%)	93 (25.76%)	868 (28.1%)	
No/Unknown	2489 (72.14%)	268 (74.24%)	2221 (71.9%)	
Systemic therapy at first record (t1), No. (%)				0.479
Intraoperative systemic and other systemic therapy before/after surgery	1 (0.03%)	0	1 (0.03%)	
Surgery both before and after systemic therapy	3 (0.09%)	0	3 (0.1%)	
Systemic therapy after surgery	598 (17.33%)	61 (16.9%)	537 (17.38%)	
Systemic therapy before surgery	63 (1.83%)	3 (0.83%)	60 (1.94%)	
Systemic therapy both before and after surgery	18 (0.52%)	4 (1.11%)	14 (0.45%)	
No systemic therapy and/or surgical procedures	2766 (80.17%)	293 (81.16%)	2473 (80.06%)	
Missing	1 (0.03%)	0	1 (0.03%)	
Pleural invasion at first record (t1), No. (%)				0.189
PL0	1909 (55.33%)	193 (53.46%)	1716 (55.55%)	
PL1 or PL2	287 (8.32%)	24 (6.65%)	263 (8.51%)	
PL3	45 (1.3%)	5 (1.39%)	40 (1.29%)	
Tumor extends to pleura but not stated if visceral or parietal	247 (7.16%)	36 (9.97%)	211 (6.83%)	

Not documented or No resection of primary or Not assessed or Unknown if assessed	962 (27.88%)	103 (28.53%)	859 (27.81%)	
Outcomes at first record (t1), No. (%)				<0.001
IPM	553 (16.03%)	155 (42.94%)	398 (12.88%)	
SPLC	2897 (83.97%)	206 (57.06%)	2691 (87.12%)	

Footnote: Values are presented as No. (%) unless otherwise indicated. P values were calculated using the chi square test. Percentages may not sum to 100 because of rounding. Abbreviations: IPM, intrapulmonary metastasis; SPLC, single primary lung cancer; NOS, not otherwise specified; PL, pleural invasion level; MLND, mediastinal lymph node dissection.

Table S4. Tumor characteristics and treatment at the second record (t2) among patients classified at the second record (t2) as IPM or SPLC.

Characteristics	Cohort N=3450	IPM N=361	SPLC N=3089	P-value
Pathology at second record (t2), No. (%)				0.007
Carcinosarcoma	1 (0.03%)	1 (0.28%)	0	
Non-small cell NEC	69 (2%)	7 (1.94%)	62 (2.01%)	
Non-small cell NET	84 (2.43%)	3 (0.83%)	81 (2.62%)	
Non-small cell adenocarcinoma	2256 (65.39%)	248 (68.7%)	2008 (65%)	
Non-small cell carcinoma	940 (27.25%)	87 (24.1%)	853 (27.61%)	
Small cell NEC	96 (2.78%)	14 (3.88%)	82 (2.65%)	
Unspecified malignant neoplasms except CNS	4 (0.12%)	1 (0.28%)	3 (0.1%)	
Tumor location at second record (t2), No. (%)				<0.001
Lower lobe	1239 (35.91%)	138 (38.23%)	1101 (35.64%)	
Middle lobe	206 (5.97%)	18 (4.99%)	188 (6.09%)	
Upper lobe	1872 (54.26%)	166 (45.98%)	1706 (55.23%)	
Main bronchus	46 (1.33%)	5 (1.39%)	41 (1.33%)	
Overlapping lesion of lung	12 (0.35%)	3 (0.83%)	9 (0.29%)	
Missing	75 (2.17%)	31 (8.59%)	44 (1.42%)	
Grade at second record (t2), No. (%)				0.063
Well-differentiated	808 (23.42%)	90 (24.93%)	718 (23.24%)	
Moderately differentiated	1441 (41.77%)	143 (39.61%)	1298 (42.02%)	
Poorly differentiated	1104 (32%)	115 (31.86%)	989 (32.02%)	
Undifferentiated	97 (2.81%)	13 (3.6%)	84 (2.72%)	
Laterality at second record (t2), No. (%)				0.024

Bilateral	1 (0.03%)	1 (0.28%)	0	
Left	1561 (45.25%)	181 (50.14%)	1380 (44.67%)	
Right	1888 (54.72%)	179 (49.58%)	1709 (55.33%)	
TNM stage at second record (t2), No. (%)				<0.001
IA	1235 (35.8%)	0	1235 (39.98%)	
IB	280 (8.12%)	0	280 (9.06%)	
IINOS	1 (0.03%)	0	1 (0.03%)	
IIA	108 (3.13%)	0	108 (3.5%)	
IIB	137 (3.97%)	105 (29.09%)	32 (1.04%)	
IIINOS	14 (0.41%)	3 (0.83%)	11 (0.36%)	
IIIA	202 (5.86%)	66 (18.28%)	136 (4.4%)	
IIIB	29 (0.84%)	7 (1.94%)	22 (0.71%)	
IV	190 (5.51%)	65 (18.01%)	125 (4.05%)	
NOS	75 (2.17%)	2 (0.55%)	73 (2.36%)	
Missing	1179 (34.17%)	113 (31.3%)	1066 (34.51%)	
T stage at second record (t2), No. (%)				<0.001
T1NOS	1 (0.03%)	0	1 (0.03%)	
T1a	1094 (31.71%)	0	1094 (35.42%)	
T1b	305 (8.84%)	0	305 (9.87%)	
T2NOS	16 (0.46%)	0	16 (0.52%)	
T2a	388 (11.25%)	0	388 (12.56%)	
T2b	45 (1.3%)	0	45 (1.46%)	
T3	216 (6.26%)	158 (43.77%)	58 (1.88%)	
T4	119 (3.45%)	90 (24.93%)	29 (0.94%)	
TX	87 (2.52%)	0	87 (2.82%)	
Missing	1179 (34.17%)	113 (31.3%)	1066 (34.51%)	
N stage at second record (t2), No. (%)				<0.001
N0	1866 (54.09%)	171 (47.37%)	1695 (54.87%)	
N1	123 (3.57%)	15 (4.16%)	108 (3.5%)	
N2	211 (6.12%)	41 (11.36%)	170 (5.5%)	
N3	39 (1.13%)	10 (2.77%)	29 (0.94%)	
NX	32 (0.93%)	11 (3.05%)	21 (0.68%)	
Missing	1179 (34.17%)	113 (31.3%)	1066 (34.51%)	
M stage at second record (t2), No. (%)				<0.001

M0	2081 (60.32%)	183 (50.69%)	1898 (61.44%)	
M1NOS	3 (0.09%)	0	3 (0.1%)	
M1a	86 (2.49%)	34 (9.42%)	52 (1.68%)	
M1b	101 (2.93%)	31 (8.59%)	70 (2.27%)	
Missing	1179 (34.17%)	113 (31.3%)	1066 (34.51%)	
Surgery at second record (t2), No. (%)				<0.001
Bronchial sleeve resection ONLY	1 (0.03%)	0	1 (0.03%)	
Electrocautery	1 (0.03%)	0	1 (0.03%)	
Excision	7 (0.2%)	2 (0.55%)	5 (0.16%)	
Excision or resection of less than one lobe	5 (0.14%)	1 (0.28%)	4 (0.13%)	
Extended pneumonectomy	1 (0.03%)	0	1 (0.03%)	
Extended pneumonectomy plus pleura or diaphragm	2 (0.06%)	0	2 (0.06%)	
Laser ablation or cryosurgery	11 (0.32%)	1 (0.28%)	10 (0.32%)	
Laser excision	5 (0.14%)	0	5 (0.16%)	
Lobe or bilobectomy extended	29 (0.84%)	5 (1.39%)	24 (0.78%)	
Lobe or bilobectomy extended WITH pericardium	1 (0.03%)	0	1 (0.03%)	
Lobe or bilobectomy extended chest wall	7 (0.2%)	3 (0.83%)	4 (0.13%)	
Lobectomy WITH mediastinal lymph node dissection	737 (21.36%)	155 (42.94%)	582 (18.84%)	
Pneumonectomy	20 (0.58%)	4 (1.11%)	16 (0.52%)	
Radiofrequency ablation	7 (0.2%)	2 (0.55%)	5 (0.16%)	
Resection of lobe or bilobectomy but less than the whole lung	172 (4.99%)	32 (8.86%)	140 (4.53%)	
Resection of lung but NOS	1 (0.03%)	0	1 (0.03%)	
Segmental resection including lingulectomy	182 (5.28%)	14 (3.88%)	168 (5.44%)	
Surgery but NOS	3 (0.09%)	1 (0.28%)	2 (0.06%)	
WITH mediastinal lymph node dissection (radical pneumonectomy)	34 (0.99%)	3 (0.83%)	31 (1%)	
Wedge resection	1035 (30%)	63 (18.84%)	967 (31.3%)	
Not-received	1183 (34.29%)	70 (19.39%)	1113 (36.03%)	
Missing	6 (0.17%)	0	6 (0.19%)	
Radiation order at second record (t2), No. (%)				0.969
Intraoperative radiation	2 (0.06%)	0	2 (0.06%)	
Radiation after surgery	235 (6.81%)	21 (5.82%)	214 (6.93%)	
Radiation before and after surgery	2 (0.06%)	0	2 (0.06%)	
Radiation prior to surgery	9 (0.26%)	1 (0.28%)	8 (0.26%)	

Sequence unknown but both were given	1 (0.03%)	0	1 (0.03%)	
Surgery both before and after radiation	1 (0.03%)	0	1 (0.03%)	
No radiation and/or cancer-directed surgery	3200 (92.75%)	339 (93.91%)	2861 (92.62%)	
Radiation type at second record (t2), No. (%)				0.586
Beam radiation	926 (26.84%)	84 (23.27%)	842 (27.26%)	
Combination of beam with implants or isotopes	1 (0.03%)	0	1 (0.03%)	
Radiation but NOS method or source not specified	11 (0.32%)	1 (0.28%)	10 (0.32%)	
Radioactive implants including brachytherapy	10 (0.29%)	0	10 (0.32%)	
Recommended, unknown if administered	33 (0.96%)	3 (0.83%)	30 (0.97%)	
None/Unknown	2450 (71.01%)	270 (74.79%)	2180 (70.57%)	
Refused	19 (0.55%)	3 (0.83%)	16 (0.52%)	
Chemotherapy at second record (t2), No. (%)				<0.001
Yes	866 (25.1%)	147 (40.72%)	719 (23.28%)	
No/Unknown	2584 (74.9%)	214 (59.28%)	2370 (76.72%)	
Systemic therapy at second record (t2), No. (%)				0.020
Intraoperative systemic and other systemic therapy before/after surgery	1 (0.03%)	0	1 (0.03%)	
Systemic therapy after surgery	462 (13.39%)	70 (19.39%)	392 (12.69%)	
Systemic therapy before surgery	16 (0.46%)	2 (0.55%)	14 (0.45%)	
Systemic therapy both before and after surgery	4 (0.12%)	0	4 (0.13%)	
No systemic therapy and/or surgical procedures	2965 (85.94%)	289 (80.06%)	2676 (86.63%)	
Missing	2 (0.06%)	0	2 (0.06%)	
Pleural invasion at second record (t2), No. (%)				0.004
PL0	1572 (45.57%)	130 (36.01%)	1442 (46.68%)	
PL1 or PL2	186 (5.39%)	20 (5.54%)	166 (5.37%)	
PL3	15 (0.43%)	2 (0.55%)	13 (0.42%)	
Tumor extends to pleura but not stated if visceral or parietal	164 (4.75%)	22 (6.09%)	142 (4.6%)	
Not documented or No resection of primary or Not assessed or Unknown if assessed	1513 (43.86%)	187 (51.8%)	1326 (42.93%)	

Footnote: Values are presented as No. (%). P values were calculated using the chi square test. Percentages may not sum to 100 because of rounding. Abbreviations: IPM, intrapulmonary metastasis; SPLC, single primary lung cancer; NEC, neuroendocrine carcinoma; NET, neuroendocrine tumor; NOS, not otherwise specified; TNM, tumor node metastasis; PL, pleural invasion level.

Table S5. Prespecified missing or registry-defined non-informative levels in candidate modeling variables among patients classified as SPLC at the first record (n = 2897).

Variable	No. with missing or non-informative level	Percent of 2897 patients	Percent of 1135 excluded patients
Pleural invasion at first record (t1)	782	26.99	68.90
T stage at first record (t1)	449	15.50	39.56
TNM stage at first record (t1)	445	15.36	39.21
N stage at first record (t1)	427	14.74	37.62
M stage at first record (t1)	413	14.26	36.39
Marital status at diagnosis (t0)	115	3.97	10.13
Tumor location at first record (t1)	24	0.83	2.11
Surgery at first record (t1)	2	0.07	0.18
Rural–urban continuum (t0)	2	0.07	0.18
Systemic therapy at first record (t1)	1	0.03	0.09
Grade at first record (t1)	0	0.00	0.00
Race at baseline (t0)	0	0.00	0.00

Footnote: Percentages in the third column are calculated using the 2897 patients classified as SPLC at the first record before complete-case exclusion. Percentages in the fourth column are calculated using the 1135 excluded patients. Counts are not mutually exclusive because a patient may have missing or non-informative levels in more than one modeling variable.

Table S6. Comparison of patients included in and excluded from the predictive modeling subset among those classified as SPLC at the first record.

Characteristics	Included in modeling subset N=1762	Excluded from modeling subset N=1135	P-value
Age phase at first record (t1), year, No. (%)			<0.001
35-39	1 (0.1)	2 (0.2)	

40-44	4 (0.2)	2 (0.2)	
45-49	31 (1.8)	13 (1.1)	
50-54	77 (4.4)	53 (4.7)	
55-59	136 (7.7)	89 (7.8)	
60-64	250 (14.2)	157 (13.8)	
65-69	430 (24.4)	210 (18.5)	
70-74	416 (23.6)	229 (20.2)	
75-79	275 (15.6)	225 (19.8)	
80-84	117 (6.6)	112 (9.9)	
>85	25 (1.4)	43 (3.8)	
Sex at baseline (t0), No. (%)			0.290
Female	965 (54.8)	598 (52.7)	
Male	797 (45.2)	537 (47.3)	
Race at baseline (t0), No. (%)			0.008
American Indian/Alaska Native	2 (0.1)	9 (0.8)	
Asian or Pacific Islander	99 (5.6)	56 (4.9)	
Black	128 (7.3)	102 (9.0)	
White	1533 (87.0)	968 (85.3)	
Marital status at diagnosis (t0), No. (%)			<0.001
Divorced	249 (14.1)	142 (12.5)	
Married (including common law)	1047 (59.4)	553 (48.7)	
Separated	23 (1.3)	15 (1.3)	
Single (never married)	186 (10.6)	111 (9.8)	
Unmarried or Domestic Partner	1 (0.1)	3 (0.3)	
Widowed	256 (14.5)	196 (17.3)	
Missing	0 (0.0)	115 (10.1)	
Median household income (t0), No. (%)			<0.001
<35,000	41 (2.3)	21 (1.9)	
35,000-39,999	63 (3.6)	39 (3.4)	
40,000-44,999	77 (4.4)	73 (6.4)	

45,000-49,999	105 (6.0)	71 (6.3)	
50,000-54,999	160 (9.1)	90 (7.9)	
55,000-59,999	125 (7.1)	102 (9.0)	
60,000-64,999	360 (20.4)	156 (13.7)	
65,000-69,999	208 (11.8)	161 (14.2)	
70,000-74,999	102 (5.8)	60 (5.3)	
>75,000	521 (29.6)	362 (31.9)	
Rural–urban continuum (t0), No. (%)			0.056
Counties in metropolitan areas of 1 million people or more	1032 (58.6)	607 (53.5)	
Counties in metropolitan areas of 250,000 to 1 million people	380 (21.6)	263 (23.2)	
Counties in metropolitan areas of less than 250 thousand people	118 (6.7)	90 (7.9)	
Nonmetropolitan counties adjacent to a metropolitan area	129 (7.3)	93 (8.2)	
Nonmetropolitan counties not adjacent to a metropolitan area	103 (5.8)	80 (7.0)	
Missing	0 (0.0)	2 (0.2)	
Pathology at first record (t1), No. (%)			<0.001
Non-small cell NEC	32 (1.8)	12 (1.1)	
Non-small cell NET	19 (1.1)	20 (1.8)	
Non-small cell adenocarcinoma	1199 (68.0)	638 (56.2)	
Non-small cell carcinoma	500 (28.4)	431 (38.0)	
Pulmonary blastoma	0 (0.0)	1 (0.1)	
Small cell NEC	10 (0.6)	31 (2.7)	
Unspecified malignant neoplasms except CNS	2 (0.1)	2 (0.2)	
Tumor location at first record (t1), No. (%)			<0.001
Lower lobe	605 (34.3)	355 (31.3)	
Middle lobe	112 (6.4)	58 (5.1)	
Upper lobe	1035 (58.7)	679 (59.8)	
Main bronchus	3 (0.2)	12 (1.1)	
Overlapping lesion of lung	7 (0.4)	7 (0.6)	
Missing	0 (0.0)	24 (2.1)	
Grade at first record (t1), No. (%)			<0.001

Well-differentiated	338 (19.2)	169 (14.9)	
Moderately differentiated	800 (45.4)	477 (42.0)	
Poorly differentiated	585 (33.2)	464 (40.9)	
Undifferentiated	39 (2.2)	25 (2.2)	
Laterality at first record (t1), No. (%)			0.151
Left	769 (43.6)	527 (46.4)	
Right	993 (56.4)	608 (53.6)	
TNM stage at first record (t1), No. (%)			<0.001
IA	863 (49.0)	293 (25.8)	
IB	478 (27.1)	111 (9.8)	
IINOS	1 (0.1)	1 (0.1)	
IIA	187 (10.6)	53 (4.7)	
IIB	81 (4.6)	29 (2.6)	
IIINOS	0 (0.0)	9 (0.8)	
IIIA	116 (6.6)	98 (8.6)	
IIIB	4 (0.2)	26 (2.3)	
IV	32 (1.8)	70 (6.2)	
Missing	0 (0.0)	445 (39.2)	
T stage at first record (t1), No. (%)			<0.001
T1NOS	0 (0.0)	1 (0.1)	
T1a	634 (36.0)	214 (18.9)	
T1b	312 (17.7)	140 (12.3)	
T2NOS	2 (0.1)	13 (1.1)	
T2a	576 (32.7)	166 (14.6)	
T2b	111 (6.3)	49 (4.3)	
T3	112 (6.4)	70 (6.2)	
T4	15 (0.9)	33 (2.9)	
Missing	0 (0.0)	448 (39.5)	
N stage at first record (t1), No. (%)			<0.001
N0	1535 (87.1)	517 (45.6)	

N1	132 (7.5)	51 (4.5)	
N2	93 (5.3)	118 (10.4)	
N3	2 (0.1)	22 (1.9)	
Missing	0 (0.0)	426 (37.5)	
M stage at first record (t1), No. (%)			<0.001
M0	1730 (98.2)	652 (57.4)	
M1a	20 (1.1)	32 (2.8)	
M1b	12 (0.7)	38 (3.3)	
Missing	0 (0.0)	412 (36.3)	
Surgery at first record (t1), No. (%)			<0.001
Bronchial sleeve resection ONLY	1 (0.1)	0 (0.0)	
Electrocautery	0 (0.0)	1 (0.1)	
Excision	0 (0.0)	3 (0.3)	
Excision or resection of less than one lobe	4 (0.2)	2 (0.2)	
Extended pneumonectomy plus pleura or diaphragm	2 (0.1)	0 (0.0)	
Laser ablation or cryosurgery	0 (0.0)	7 (0.6)	
Laser excision	0 (0.0)	1 (0.1)	
Lobe or bilobectomy extended	25 (1.4)	13 (1.1)	
Lobe or bilobectomy extended WITH diaphragm	1 (0.1)	0 (0.0)	
Lobe or bilobectomy extended WITH pericardium	1 (0.1)	0 (0.0)	
Lobe or bilobectomy extended chest wall	8 (0.5)	3 (0.3)	
Lobectomy WITH mediastinal lymph node dissection	955 (54.2)	298 (26.3)	
Pneumonectomy	13 (0.7)	1 (0.1)	
Radiofrequency ablation	0 (0.0)	1 (0.1)	
Resection of lobe or bilobectomy but less than the whole lung	178 (10.1)	61 (5.4)	
Resection of lung but NOS	0 (0.0)	2 (0.2)	
Segmental resection including lingulectomy	104 (5.9)	36 (3.2)	
Surgery but NOS	0 (0.0)	2 (0.2)	
WITH mediastinal lymph node dissection (radical pneumonectomy)	17 (1.0)	11 (1.0)	

Wedge resection	349 (19.8)	124 (10.9)	
Not-received	104 (5.9)	567 (50.0)	
Missing	0 (0.0)	2 (0.2)	
Radiation order at first record (t1), No. (%)			0.154
Radiation after surgery	105 (6.0)	86 (7.6)	
Radiation before and after surgery	3 (0.2)	0 (0.0)	
Radiation prior to surgery	13 (0.7)	9 (0.8)	
Sequence unknown but both were given	2 (0.1)	0 (0.0)	
Surgery both before and after radiation	3 (0.2)	0 (0.0)	
No radiation and/or cancer-directed surgery	1636 (92.8)	1040 (91.6)	
Radiation type at first record (t1), No. (%)			<0.001
Beam radiation	137 (7.8)	430 (37.9)	
Radiation but NOS method or source not specified	4 (0.2)	4 (0.4)	
Radioactive implants including brachytherapy	4 (0.2)	0 (0.0)	
Radioisotopes	8 (0.5)	17 (1.5)	
Recommended, unknown if administered	4 (0.2)	4 (0.4)	
None/Unknown	1601 (90.9)	681 (60.0)	
Refused	8 (0.5)	3 (0.3)	
Chemotherapy at first record (t1), No. (%)			<0.001
Yes	372 (21.1)	377 (33.2)	
No/Unknown	1390 (78.9)	758 (66.8)	
Systemic therapy at first record (t1), No. (%)			0.018
Surgery both before and after systemic therapy	2 (0.1)	1 (0.1)	
Systemic therapy after surgery	312 (17.7)	152 (13.4)	
Systemic therapy before surgery	35 (2.0)	20 (1.8)	
Systemic therapy both before and after surgery	11 (0.6)	3 (0.3)	
No systemic therapy and/or surgical procedures	1402 (79.6)	958 (84.4)	
Missing	0 (0.0)	1 (0.1)	
Pleural invasion at first record (t1), No. (%)			<0.001
PL0	1382 (78.4)	269 (23.7)	

PL1 or PL2	183 (10.4)	48 (4.2)
PL3	35 (2.0)	6 (0.5)
Tumor extends to pleura but not stated if visceral or parietal	162 (9.2)	30 (2.6)
Not documented or No resection of primary or Not assessed or Unknown if assessed	0 (0.0)	782 (68.9)

Abbreviations: IPM, intrapulmonary metastasis; SPLC, single primary lung cancer; SD, standard deviation; NEC, neuroendocrine carcinoma; NET, neuroendocrine tumor; CNS, central nervous system; NOS, not otherwise specified; TNM, tumor node metastasis; PL, pleural invasion level.

Table S7. Comparison of predictor distributions between the training cohort and the temporal test cohort for the random forest analysis.

Predictor	Variable type	Training cohort	Temporal test cohort	Levels retained	Between period difference
Sex at baseline (t0)	Binary	Male, 628 (46.6%)	Male, 169 (40.8%)	—	SMD = 0.116
Chemotherapy at first record (t1)	Binary	Yes, 285 (21.1%)	Yes, 87 (21.0%)	—	SMD = 0.003
Time_Interval_1	Numeric	2.080 ± 1.979; median 2.000 [0.000, 4.000]	0.836 ± 1.002; median 0.000 [0.000, 2.000]	—	SMD = 0.793
Surgery at first record (t1)	Categorical	Overall distribution compared across levels	Overall distribution compared across levels	9	Max absolute standardized difference = 0.153
Systemic therapy at first record (t1)	Categorical	Overall distribution compared across levels	Overall distribution compared across levels	5	Max absolute standardized difference = 0.134
Age phase at first record (t1)	Categorical	Overall distribution compared across levels	Overall distribution compared across levels	11	Max absolute standardized difference = 0.104

Marital status at diagnosis (t0)	Categorical	Overall distribution compared across levels	Overall distribution compared across levels	6	Max absolute standardized difference = 0.103
Pleural invasion at first record (t1)	Categorical	Overall distribution compared across levels	Overall distribution compared across levels	4	Max absolute standardized difference = 0.103
Median household income (t0)	Categorical	Overall distribution compared across levels	Overall distribution compared across levels	10	Max absolute standardized difference = 0.100
T stage at first record (t1)	Categorical	Overall distribution compared across levels	Overall distribution compared across levels	7	Max absolute standardized difference = 0.099
Pathology at first record (t1)	Categorical	Overall distribution compared across levels	Overall distribution compared across levels	6	Max absolute standardized difference = 0.096
Radiation order at first record (t1)	Categorical	Overall distribution compared across levels	Overall distribution compared across levels	4	Max absolute standardized difference = 0.081
M stage at first record (t1)	Categorical	Overall distribution compared across levels	Overall distribution compared across levels	3	Max absolute standardized difference = 0.079
N stage at first record (t1)	Categorical	Overall distribution compared across levels	Overall distribution compared across levels	4	Max absolute standardized difference = 0.075
Rural-urban continuum (t0)	Categorical	Overall distribution compared across levels	Overall distribution compared across levels	5	Max absolute standardized difference = 0.072

TNM stage at first record (t1)	Categorical	Overall distribution compared across levels	Overall distribution compared across levels	8	Max absolute standardized difference = 0.070
Location at first record (t1)	Categorical	Overall distribution compared across levels	Overall distribution compared across levels	4	Max absolute standardized difference = 0.065
Race at baseline (t0)	Categorical	Overall distribution compared across levels	Overall distribution compared across levels	4	Max absolute standardized difference = 0.055
Laterality at first record (t1)	Categorical	Overall distribution compared across levels	Overall distribution compared across levels	2	Max absolute standardized difference = 0.053
Radiation type at first record (t1)	Categorical	Overall distribution compared across levels	Overall distribution compared across levels	3	Max absolute standardized difference = 0.047
Grade at first record (t1)	Categorical	Overall distribution compared across levels	Overall distribution compared across levels	4	Max absolute standardized difference = 0.026

Footnote: The training cohort included patients whose first record occurred from 2000 to 2013, and the temporal test cohort included patients whose first record occurred from 2014 to 2019. Binary predictors are presented as the number and percentage of patients in the positive category shown. The numeric predictor is presented as mean ± standard deviation and median with interquartile range. For binary and numeric predictors, between period differences are summarized using the standardized mean difference. For categorical predictors, “Levels retained” indicates the number of analyzed categories after preprocessing, and between period difference is summarized as the maximum absolute standardized difference across levels. Larger absolute values indicate greater distributional differences between periods. Abbreviations: IQR, interquartile range; SMD, standardized mean difference; TNM, tumor node metastasis.

Table S8. Extended performance metrics of the random forest model in the internal training analysis and temporal test cohort.

Metric	Internal training analysis	Temporal test cohort
Cohort size, n	1348	414
IPM events, n	122	21
IPM prevalence, %	9.1	5.1
Accuracy	0.935	0.973
IPM precision	0.72	1
IPM recall	0.48	0.48
IPM F1 score	0.57	0.65
Decision threshold	0.34	0.34
AUC	0.852	0.929
95% CI for AUC	0.813 to 0.890	0.861 to 0.982
Average precision	0.592	0.7
Brier score	0.053	0.024
Null Brier	0.082	0.048
Brier skill score	0.352	0.503
Calibration intercept	-0.026	-0.097
Calibration slope	0.873	1.57

Footnote: Internal performance was estimated using nested cross validation with out of fold predictions within the training cohort. Temporal performance was evaluated by applying the final model trained on the full development cohort to the temporal test cohort. The decision threshold was selected in the training cohort by maximizing F1 and then applied unchanged to the temporal test cohort. Null Brier denotes the Brier score of a prevalence only reference model, and Brier skill score was calculated relative to this null model. Because the temporal test cohort contained only 21 IPM events, performance estimates in that cohort, particularly calibration related measures, should be interpreted cautiously. Abbreviations: AUC, area under the curve; CI, confidence interval; IPM, intrapulmonary metastasis.

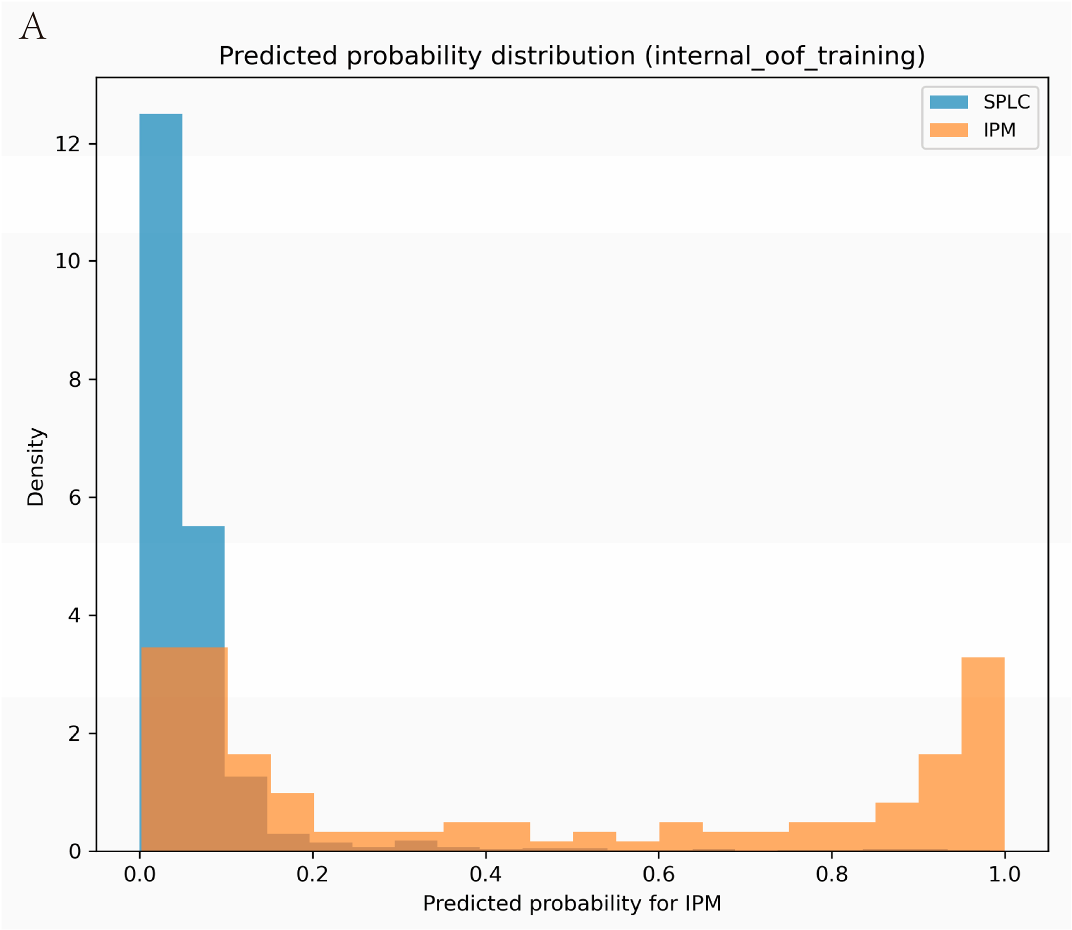
A
Predicted probability distribution (internal_oof_training)
SPLC
IPM
Density
Predicted probability for IPM
0
2
4
6
8
10
12
0.0
0.2
0.4
0.6
0.8
1.0

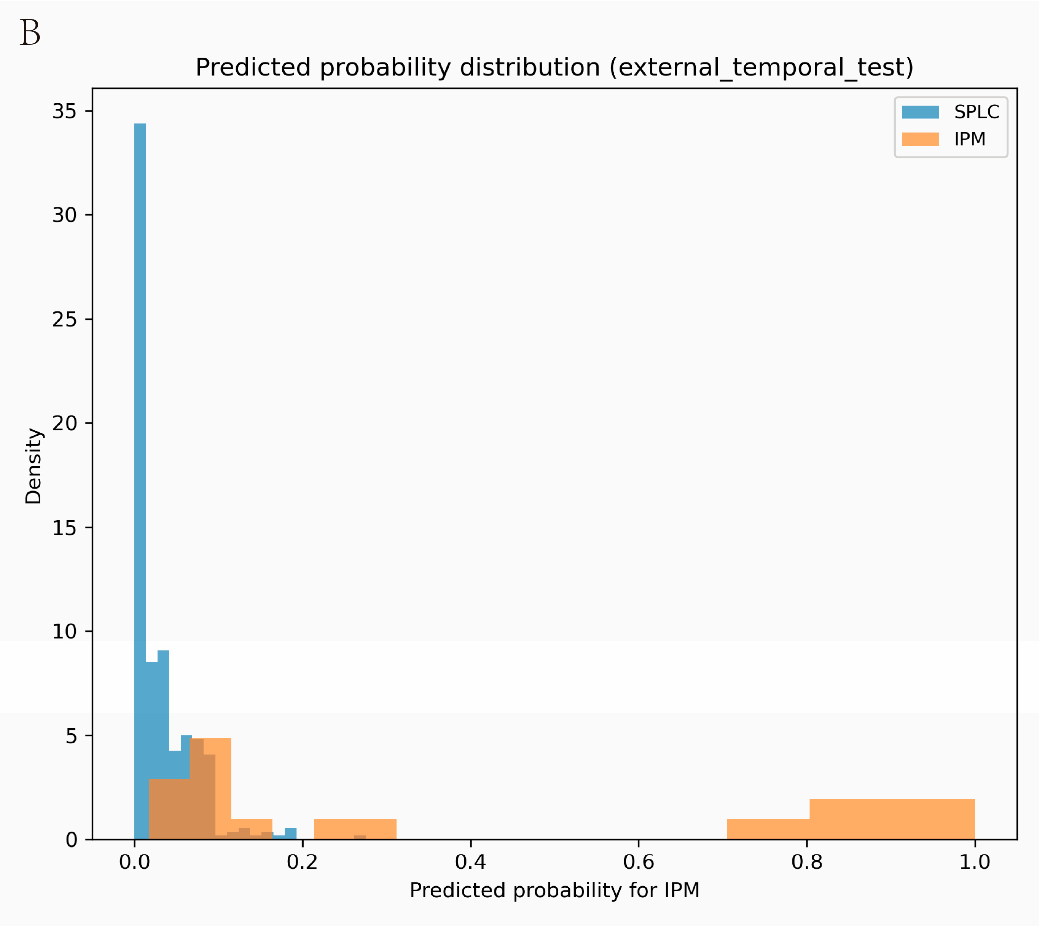

Figure S2. Distribution of predicted probabilities for IPM generated by the random forest model in the internal training analysis and the temporal validation cohort. (A) In the internal training analysis, probabilities were based on nested cross validation out of fold predictions. (B) In the temporal validation cohort, probabilities were generated by applying the final calibrated model trained on the full development cohort to later years. Abbreviations: IPM, intrapulmonary metastasis; SPLC, single primary lung cancer.

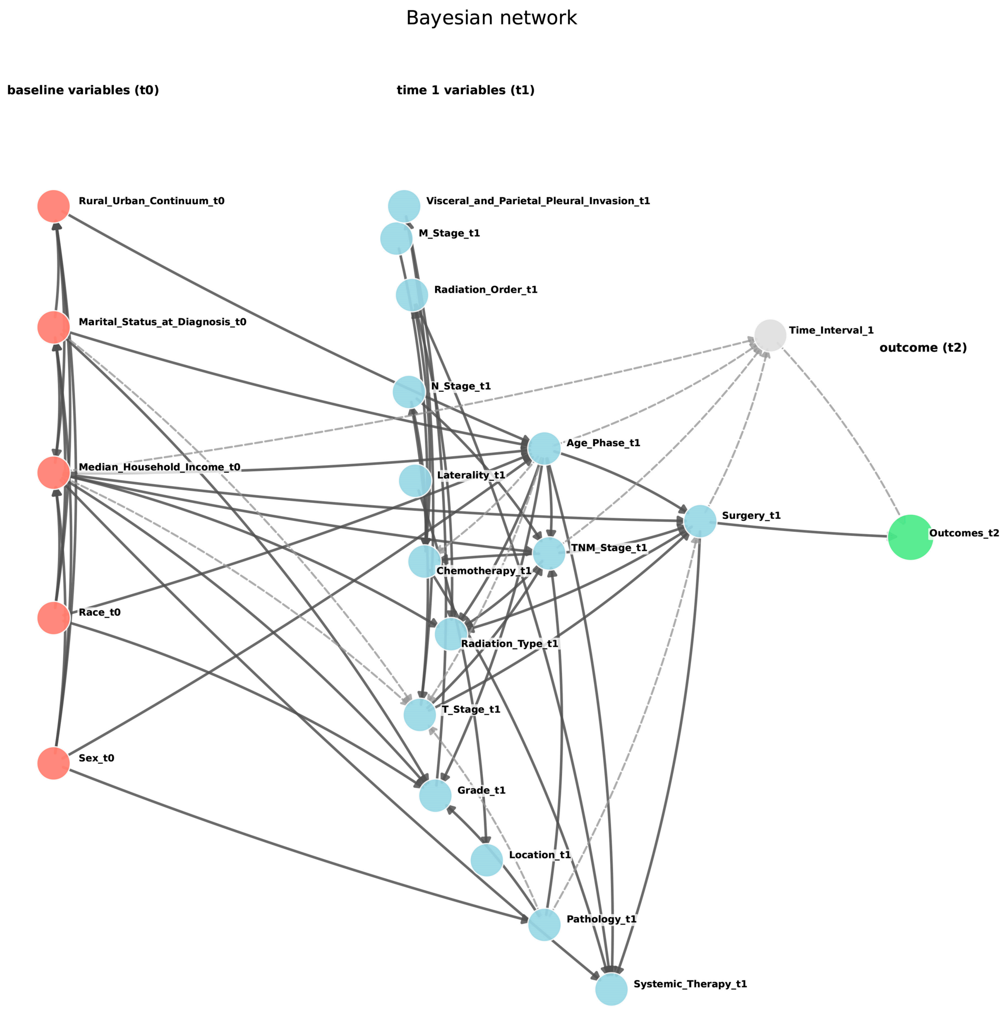
Bayesian network
baseline variables (t0)
time 1 variables (t1)
outcome (t2)
Rural_Urban_Continuum_t0
Marital_Status_at_Diagnosis_t0
Median_Household_Income_t0
Race_t0
Sex_t0
Visceral_and_Parietal_Pleural_Invasion_t1
M_Stage_t1
Radiation_Order_t1
N_Stage_t1
Age_Phase_t1
Laterality_t1
Chemotherapy_t1
TNM_Stage_t1
Radiation_Type_t1
T_Stage_t1
Grade_t1
Location_t1
Pathology_t1
Systemic_Therapy_t1
Surgery_t1
Time_Interval_1
Outcomes_t2

Figure S3. Extended Bayesian network including all arcs with bootstrap frequency ≥ 0.50. Nodes are arranged by temporal layer. Pink nodes indicate baseline variables at t0, light blue nodes indicate variables at t1, and the light green node indicates the outcome at t2. Solid arrows represent the directed dependencies shown in the learned network.

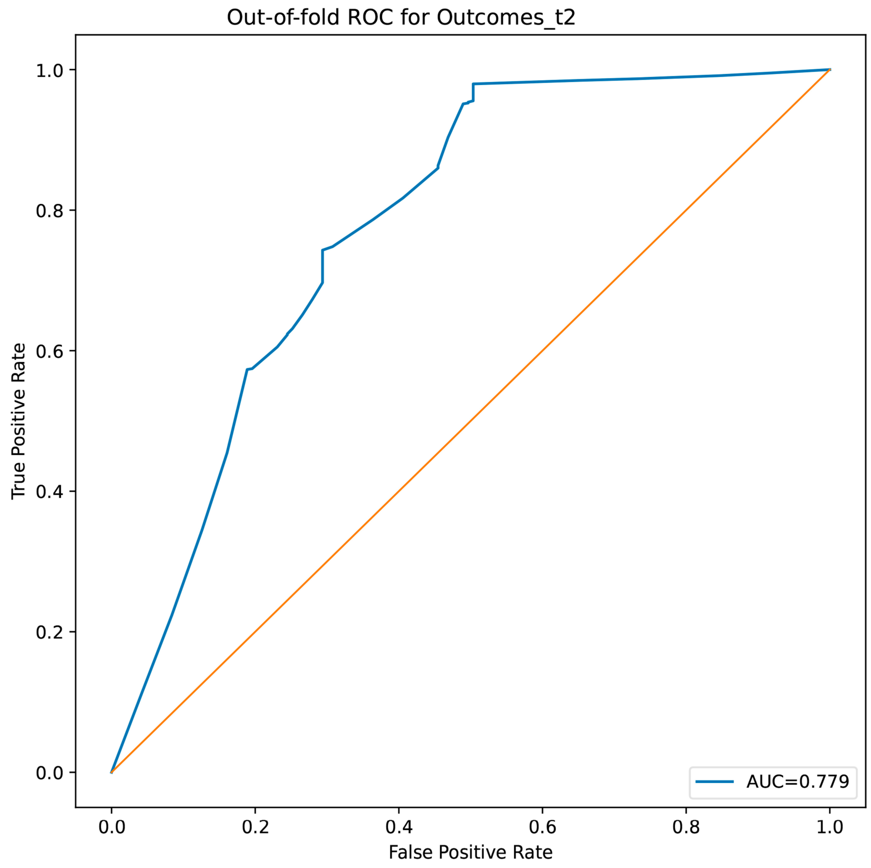

Figure S4. ROC curves for predicting Outcomes_t2 using forward chaining evaluation. Abbreviations: ROC, Receiver operating characteristic.

Variable	SI	Outcome_t2	P	RR (95% CI)
Sex_t0	Female -> Male	IPM	0.266	0.980 (0.943 to 1.016)
	Female -> Male	SPLC	0.266	1.002 (0.999 to 1.006)
Race_t0	Black -> White	IPM	0.932	1.003 (0.938 to 1.073)
Race_t0	Black -> White	SPLC	0.932	1.000 (0.992 to 1.006)
Race_t0	Asian or Pacific Islander -> White	IPM	0.910	0.994 (0.908 to 1.103)
Race_t0	Asian or Pacific Islander -> White	SPLC	0.910	1.001 (0.991 to 1.010)
Age_Phase_t1	> 60 -> < 60	IPM	0.398	0.893 (0.739 to 1.159)
	> 60 -> < 60	SPLC	0.398	1.013 (0.988 to 1.049)
Grade_t1	Well differentiated -> Undifferentiated	IPM	0.186	0.982 (0.937 to 1.006)
	Well differentiated -> Undifferentiated	SPLC	0.186	1.002 (0.999 to 1.007)
Grade_t1	Moderately differentiated -> Undifferentiated	IPM	0.342	0.989 (0.947 to 1.010)
	Moderately differentiated -> Undifferentiated	SPLC	0.342	1.001 (0.999 to 1.006)
Grade_t1	Poorly differentiated -> Undifferentiated	IPM	0.710	0.995 (0.956 to 1.019)
	Poorly differentiated -> Undifferentiated	SPLC	0.710	1.000 (0.998 to 1.005)
Grade_t1	Well differentiated -> Poorly differentiated	IPM	0.208	0.986 (0.965 to 1.008)
	Well differentiated -> Poorly differentiated	SPLC	0.208	1.001 (0.999 to 1.004)
Grade_t1	Well differentiated -> Moderately differentiated	IPM	0.340	0.993 (0.979 to 1.007)
	Well differentiated -> Moderately differentiated	SPLC	0.340	1.001 (0.999 to 1.002)
Pathology_t1	Non−small cell NEC -> NSCA	IPM	0.132	1.297 (0.968 to 1.665)
	Non−small cell NEC -> NSCA	SPLC	0.132	0.972 (0.927 to 1.003)
Pathology_t1	Non−small cell NET -> NSCA	IPM	0.148	1.304 (0.950 to 1.716)
	Non−small cell NET -> NSCA	SPLC	0.148	0.971 (0.922 to 1.004)
Pathology_t1	Non−small cell carcinoma -> NSCA	IPM	0.120	1.079 (0.985 to 1.176)
	Non−small cell carcinoma -> NSCA	SPLC	0.120	0.992 (0.981 to 1.001)
Pathology_t1	Small cell NEC -> NSCA	IPM	0.160	1.331 (0.951 to 1.768)
	Small cell NEC -> NSCA	SPLC	0.160	0.969 (0.917 to 1.004)
TNM_Stage_t1	II, III, and IV-> I	IPM	0.106	1.152 (0.985 to 1.279)
	II, III, and IV-> I	SPLC	0.106	0.982 (0.953 to 1.001)
TNM_Stage_t1	III and IV-> I and II	IPM	0.100	1.058 (0.991 to 1.096)
	III and IV-> I and II	SPLC	0.100	0.993 (0.981 to 1.001)
TNM_Stage_t1	IV-> I, II, and III	IPM	0.566	1.017 (0.967 to 1.085)
	IV-> I, II, and III	SPLC	0.566	0.998 (0.988 to 1.004)

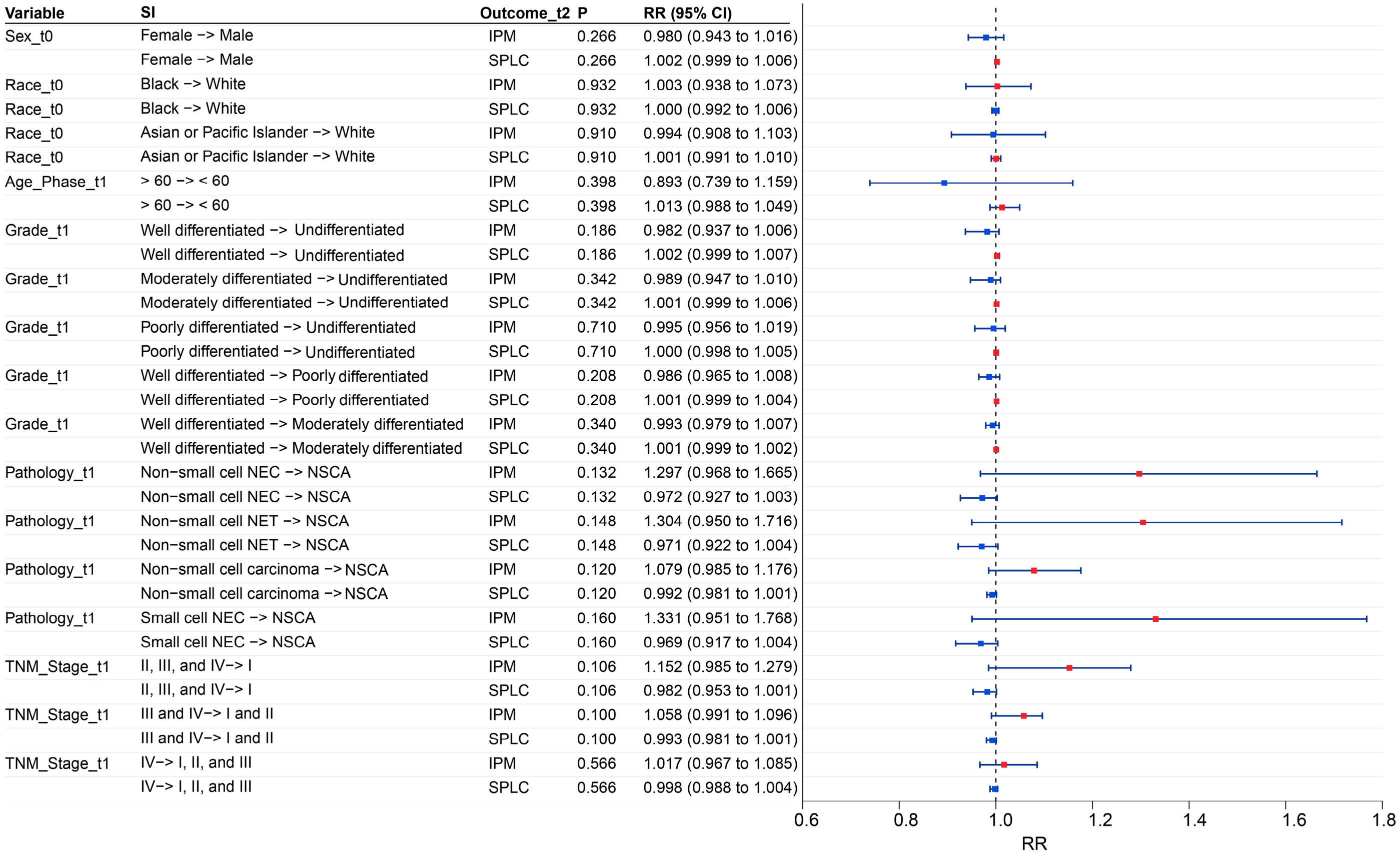

Figure S5. Model-implied RRs for Outcomes_t2 under SIs on selected clinicopathological variables at t1. Forest plots show RRs and 95% CIs derived from bootstrap resampling. Abbreviations: RR, risk ratio; SI, simulated intervention; CI, confidence interval; NSCA, Non-small cell adenocarcinoma.

Table S9. Overall bounds summary for subsequent IPM classification among patients with initial SPLC.

Total patients	Observed outcomes, n	Missing outcomes, n	Missing fraction, %	Observed IPM, n	Observed IPM rate, %	Lower bound, %	Upper bound, %
37700	2897	34803	92.3	206	7.11	0.55	92.86

Footnote: Patients were restricted to those classified as SPLC at t1. Observed outcomes refer to patients with a second record and thus observable t2 classification. Missing outcomes refer to patients without a second record. The lower bound assumes that all patients without a second record were not classified as IPM at t2, whereas the upper bound assumes that all such patients were classified as IPM. Abbreviations: IPM, intrapulmonary metastasis; SPLC, single primary lung cancer.

Table S10. Overall scenario analysis for subsequent IPM classification among patients with initial SPLC.

Scenario type	Assumption	Overall IPM rate, %
Fixed probability	Missing cases assigned IPM probability of 0%	0.55
Fixed probability	Missing cases assigned IPM probability of 1%	1.47
Fixed probability	Missing cases assigned IPM probability of 3%	3.32
Fixed probability	Missing cases assigned IPM probability of 5%	5.16
Fixed probability	Missing cases assigned IPM probability of 10%	9.78
Fixed probability	Missing cases assigned IPM probability of 20%	19.01
Multiplier	Missing case IPM probability set to 0.25 × observed IPM rate	2.19
Multiplier	Missing case IPM probability set to 0.50 × observed IPM rate	3.83
Multiplier	Missing case IPM probability set to 0.75 × observed IPM rate	5.47
Multiplier	Missing case IPM probability set to 1.00 × observed IPM rate	7.11
Multiplier	Missing case IPM probability set to 1.25 × observed IPM rate	8.75
Multiplier	Missing case IPM probability set to 1.50 × observed IPM rate	10.39
Multiplier	Missing case IPM probability set to 2.00 × observed IPM rate	13.68

Footnote: Patients were restricted to those classified as SPLC at t1. In the fixed probability scenarios, each patient without a second record was assigned the same prespecified probability of IPM. In the multiplier scenarios, the IPM probability among patients without a second record was set to a multiple of the observed IPM rate among patients with a second record. Abbreviations: IPM, intrapulmonary metastasis; SPLC, single primary lung cancer.

Table S11. Stratified bounds and fixed probability scenario analysis by surgery at t1 among patients with initial SPLC.

Surgery at t1	Total, n	Observed, n	Missing, n	Missing, %	Observed IPM, n	Observed IPM rate, %	Lower bound, %	Upper bound, %	Scenario 1%, %	Scenario 3%, %	Scenario 5%, %	Scenario 10%, %	Scenario 20%, %
Not received	14858	671	14187	95.5	110	16.39	0.74	96.22	1.7	3.6	5.51	10.29	19.84
Laser ablation or cryosurgery	29	7	22	75.9	1	14.29	3.45	79.31	4.21	5.72	7.24	11.03	18.62
Radical pneumonectomy WITH MLND	412	28	384	93.2	3	10.71	0.73	93.93	1.66	3.52	5.39	10.05	19.37
Wedge resection	3170	473	2697	85.1	38	8.03	1.2	86.28	2.05	3.75	5.45	9.71	18.21
Lobe or bilobectomy extended	38	38	0	0	3	7.89	7.89	7.89	7.89	7.89	7.89	7.89	7.89
Pneumonectomy	154	14	140	90.9	1	7.14	0.65	91.56	1.56	3.38	5.19	9.74	18.83
Segmental resection including lingulectomy	949	140	809	85.2	6	4.29	0.63	85.88	1.48	3.19	4.89	9.16	17.68
Resection of lobe or bilobectomy but less than the whole lung	3031	239	2792	92.1	10	4.18	0.33	92.44	1.25	3.09	4.94	9.54	18.75
Lobectomy WITH MLND	14134	1253	12881	91.1	33	2.63	0.23	91.37	1.14	2.97	4.79	9.35	18.46
Excision or resection of less than one lobe	64	6	58	90.6	0	0	0	90.62	0.91	2.72	4.53	9.06	18.12
Lobe or bilobectomy extended chest wall	11	11	0	0	0	0	0	0	0	0	0	0	0

Footnote: Patients were restricted to those classified as SPLC at t1. Surgery categories were harmonized before analysis. Observed outcomes refer to patients with a second record. Missing outcomes refer to patients without a second record. Lower and upper bounds were defined as

in Table S9. Scenario columns show the overall IPM rate within each surgery stratum under fixed assumed probabilities of IPM among patients without a second record. Categories with no missing outcomes yield identical values across scenario assumptions. Small strata should be interpreted cautiously. Abbreviations: IPM, intrapulmonary metastasis; SPLC, single primary lung cancer; MLND, mediastinal lymph node dissection.

Table S12. Stratified multiplier scenario analysis by surgery at t1 among patients with initial SPLC.

Surgery at t1	Observed IPM rate, %	0.25 × observed, %	0.50 × observed, %	0.75 × observed, %	1.00 × observed, %	1.25 × observed, %	1.50 × observed, %	2.00 × observed, %
Not received	16.39	4.65	8.57	12.48	16.39	20.31	24.22	32.05
Laser ablation or cryosurgery	14.29	6.16	8.87	11.58	14.29	17	19.7	25.12
Radical pneumonectomy WITH MLND	10.71	3.22	5.72	8.22	10.71	13.21	15.71	20.7
Wedge resection	8.03	2.91	4.62	6.33	8.03	9.74	11.45	14.87
Lobe or bilobectomy extended	7.89	7.89	7.89	7.89	7.89	7.89	7.89	7.89
Pneumonectomy	7.14	2.27	3.9	5.52	7.14	8.77	10.39	13.64
Segmental resection including lingulectomy	4.29	1.55	2.46	3.37	4.29	5.2	6.11	7.94
Resection of lobe or bilobectomy but less than the whole lung	4.18	1.29	2.26	3.22	4.18	5.15	6.11	8.04
Lobectomy WITH MLND	2.63	0.83	1.43	2.03	2.63	3.23	3.83	5.03
Excision or resection of less than one lobe	0	0	0	0	0	0	0	0
Lobe or bilobectomy extended chest wall	0	0	0	0	0	0	0	0

Footnote: Patients were restricted to those classified as SPLC at t1. Surgery categories were harmonized before analysis. The assumed IPM probability among patients without a second record was set to a multiple of the observed within stratum IPM rate. Categories with no missing outcomes yield identical values across multiplier assumptions. Small strata should be interpreted cautiously. Abbreviations: IPM, intrapulmonary metastasis; SPLC, single primary lung cancer; MLND, mediastinal lymph node dissection.

Table S13. Stratified bounds and fixed probability scenario analysis by pleural invasion at t1 among patients with initial SPLC.

Pleural invasion at t1	Total, n	Observed, n	Missing, n	Missing, %	Observed IPM, n	Observed IPM rate, %	Lower bound, %	Upper bound, %	Scenario 1%, %	Scenario 3%, %	Scenario 5%, %	Scenario 10%, %	Scenario 20%, %
Tumor extends to pleura, NOS	2524	192	2332	92.4	22	11.46	0.87	93.26	1.8	3.64	5.49	10.11	19.35
PL3	378	41	337	89.2	4	9.76	1.06	90.21	1.95	3.73	5.52	9.97	18.89
PL1 or PL2	2556	231	2325	91	9	3.9	0.35	91.31	1.26	3.08	4.9	9.45	18.54
PL0	16160	1651	14509	89.8	122	7.39	0.75	90.54	1.65	3.45	5.24	9.73	18.71
Unknown or not assessed	16082	782	15300	95.1	49	6.27	0.3	95.44	1.26	3.16	5.06	9.82	19.33

Footnote: Patients were restricted to those classified as SPLC at t1. Pleural invasion categories were harmonized before analysis into PL0, PL1 or PL2, PL3, tumor extends to pleura not otherwise specified, and unknown or not assessed. Observed outcomes refer to patients with a second record. Missing outcomes refer to patients without a second record. Lower and upper bounds were defined as in Table S9. Scenario columns show the overall IPM rate within each pleural invasion stratum under fixed assumed probabilities of IPM among patients without a second record. Small strata should be interpreted cautiously. Abbreviations: IPM, intrapulmonary metastasis; NOS, not otherwise specified; SPLC, single primary lung cancer.

Table S14. Stratified multiplier scenario analysis by pleural invasion at t1 among patients with initial SPLC.

Pleural invasion at t1	Observed IPM rate, %	0.25 × observed, %	0.50 × observed, %	0.75 × observed, %	1.00 × observed, %	1.25 × observed, %	1.50 × observed, %	2.00 × observed, %
Tumor extends to pleura, NOS	11.46	3.52	6.16	8.81	11.46	14.11	16.75	22.05
PL3	9.76	3.23	5.41	7.58	9.76	11.93	14.11	18.45
PL1 or PL2	3.9	1.24	2.12	3.01	3.9	4.78	5.67	7.44
PL0	7.39	2.41	4.07	5.73	7.39	9.05	10.71	14.02
Unknown or not assessed	6.27	1.8	3.29	4.78	6.27	7.76	9.25	12.23

Footnote: Patients were restricted to those classified as SPLC at t1. Pleural invasion categories were harmonized before analysis. The assumed IPM probability among patients without a second record was set to a multiple of the observed within stratum IPM rate. Small strata should be interpreted cautiously. Abbreviations: IPM, intrapulmonary metastasis; NOS, not otherwise specified; SPLC, single primary lung cancer.
